# Changes in primary care visits for respiratory illness during the COVID-19 pandemic: a multinational study by the International Consortium of Primary Care Big Data Researchers (INTRePID)

**DOI:** 10.3389/fmed.2024.1343646

**Published:** 2024-06-17

**Authors:** John M. Westfall, Angela Ortigoza Bonilla, María C. Lapadula, Paula L. Zingoni, William C. W. Wong, Knut A. Wensaas, Wilson D. Pace, Javier Silva-Valencia, Luciano F. Scattini, Amy P. P. Ng, Jo-Anne Manski-Nankervis, Zheng J. Ling, Zhuo Li, Adrian H. Heald, Adrian Laughlin, Robert S. Kristiansson, Christine M. Hallinan, Lay H. Goh, Gabriela Gaona, Signe Flottorp, Simon de Lusignan, María S. Cuba-Fuentes, Valborg Baste, Karen Tu, on behalf of INTRePID

**Affiliations:** ^1^DARTNet Institute, Aurora, CO, United States; ^2^Department of Family and Community Medicine, University of Toronto, Toronto, ON, Canada; ^3^Ministry of Health of the Autonomous City of Buenos Aires, Buenos Aires, Argentina; ^4^Department of Family Medicine and Primary Care, The University of Hong Kong-Shenzhen Hospital, Shenzhen, China; ^5^Department of Family Medicine and Primary Care, School of Clinical Medicine, Li Ka Shing Faculty of Medicine, The University of Hong Kong, Hong Kong, China; ^6^Research Unit for General Practice, NORCE Norwegian Research Centre AS, Bergen, Norway; ^7^Center for Research in Primary Health Care (CINAPS), Universidad Peruana Cayetano Heredia, Lima, Peru; ^8^North York General Hospital, Toronto, ON, Canada; ^9^Department of General Practice and Primary Care, The University of Melbourne, Melbourne, VIC, Australia; ^10^Division of Family Medicine, Yong Loo Lin School of Medicine, National University of Singapore, Singapore, Singapore; ^11^School of Medical Sciences, Division of Diabetes, Endocrinology and Gastroenterology, University of Manchester, Manchester, United Kingdom; ^12^Department of Public Health and Caring Sciences, Uppsala University, Uppsala, Sweden; ^13^Centre for Epidemic Interventions Research, Norwegian Institute of Public Health, Oslo, Norway; ^14^Department of General Practice, University of Oslo, Oslo, Norway; ^15^Nuffield Department of Primary Care Health Sciences, University of Oxford, Oxford, United Kingdom; ^16^National Centre for Emergency Primary Health Care, NORCE Norwegian Research Centre, Bergen, Norway; ^17^Departments of Research and Innovation and Family Medicine-North York General Hospital, Toronto Western Family Health Team-University Health Network, Toronto, ON, Canada

**Keywords:** COVID-19, acute respiratory illness, chronic respiratory illness, primary care, asthma, COPD, reason for visit, international comparison

## Abstract

**Objectives:**

The majority of patients with respiratory illness are seen in primary care settings. Given COVID-19 is predominantly a respiratory illness, the INTernational ConsoRtium of Primary Care BIg Data Researchers (INTRePID), assessed the pandemic impact on primary care visits for respiratory illnesses.

**Design:**

Definitions for respiratory illness types were agreed on collectively. Monthly visit counts with diagnosis were shared centrally for analysis.

**Setting:**

Primary care settings in Argentina, Australia, Canada, China, Norway, Peru, Singapore, Sweden and the United States.

**Participants:**

Over 38 million patients seen in primary care settings in INTRePID countries before and during the pandemic, from January 1st, 2018, to December 31st, 2021.

**Main outcome measures:**

Relative change in the monthly mean number of visits before and after the onset of the pandemic for acute infectious respiratory disease visits including influenza, upper and lower respiratory tract infections and chronic respiratory disease visits including asthma, chronic obstructive pulmonary disease, respiratory allergies, and other respiratory diseases.

**Results:**

INTRePID countries reported a marked decrease in the average monthly visits for respiratory illness. Changes in visits varied from −10.9% [95% confidence interval (CI): −33.1 to +11.3%] in Norway to −79.9% (95% CI: −86.4% to −73.4%) in China for acute infectious respiratory disease visits and − 2.1% (95% CI: −12.1 to +7.8%) in Peru to −59.9% (95% CI: −68.6% to −51.3%) in China for chronic respiratory illness visits. While seasonal variation in allergic respiratory illness continued during the pandemic, there was essentially no spike in influenza illness during the first 2 years of the pandemic.

**Conclusion:**

The COVID-19 pandemic had a major impact on primary care visits for respiratory presentations. Primary care continued to provide services for respiratory illness, although there was a decrease in infectious illness during the COVID pandemic. Understanding the role of primary care may provide valuable information for COVID-19 recovery efforts and planning for future global emergencies.

## 1 Introduction

In the 3 years since the World Health Organization (WHO) declared COVID-19 a global pandemic, there have been over 659 million cases with over 6.5 million deaths worldwide (1, 2). The COVID-19 pandemic has presented unprecedented challenges to primary health care globally. Governments have implemented policies to prioritize using healthcare resources to treat COVID-19 patients and prevent the spread of the disease, such as quarantines, virtual work/school, wearing a mask, and social distancing. Although changes occurred rapidly in response to the COVID-19 pandemic (3), we have not yet determined the implications of these changes for other respiratory diseases. Numerous papers describe the potential and real impact of COVID-19 on primary care (4, 5). Huston et al. described the role of primary care in triaging and treating patients with COVID-19 in six well-resourced countries, including Australia, New Zealand, Canada, the Netherlands, the United Kingdom, and the United States (6). They discussed the negative impact of COVID-19 on access to primary care, the stress of decreased patient encounters on financial viability, and the capacity of primary care to respond to such a widespread pandemic. Research has identified socioeconomic disadvantage as an independent risk factor for death following a COVID-19 infection in individuals with type 2 diabetes (7) and those with other long-term conditions (8). There was wide variation in COVID-19 mortality rate between countries (9).

Upper respiratory illness is one of the most common diagnoses seen in primary care, accounting for 5–20% of ambulatory visits (10–13). Each year, in the United States alone, 20 million people with respiratory illnesses account for 64 million visits to primary care (14). Primary care serves over 90% of patients with lower respiratory illness or pneumonia. Since COVID-19 is predominantly a respiratory illness, primary care practices have been on the front lines of care for COVID-19 patients. Their role includes diagnosis, triage to the appropriate level of care, supportive care, treatments, and immunizations since they became available. While primary care has continued to provide in-person and virtual visits throughout the pandemic, the impact of COVID-19 on primary care visits for respiratory illness is unknown.

The INTernational ConsoRtium of Primary Care BIg Data Researchers (INTRePID) began as a collaboration between primary care researchers across the globe in response to the COVID-19 pandemic (15). INTRePID participants provide de-identified aggregated electronic data from electronic health records and billing claims data. Data are harmonized and analyzed centrally at the University of Toronto Department of Family and Community Medicine.

While respiratory infection is among the most common diagnoses in primary care, there is limited evidence of the impact of COVID-19 on respiratory illness care in primary care (12). The purpose of this paper is to describe the international experience of primary care practices related to respiratory illness before and during the COVID-19 pandemic.

## 2 Materials and methods

### 2.1 Study design

This study employed a retrospective observational design to investigate the impact of the COVID-19 pandemic on primary care visits for respiratory illnesses across 9 countries. The study period spanned from January 1, 2018, to December 31, 2021.

### 2.2 Data sources

Data for this study were gathered from diverse sources, including electronic medical records and billing claims. Specifically, information was sourced from visits to primary care physicians in Australia, Canada, China, Norway, Singapore, Sweden, and the United States. Moreover, data were obtained from primary care clinics in Argentina and Peru, encompassing visits to various healthcare providers within a primary care setting.

The dataset covers the period from January 1, 2018, to December 31, 2021, with the exception of Peru, where visit data was available only from January 2019. Although the onset of the pandemic varied across countries, for the purpose of this study, we defined the pre-pandemic period as January 2018 to March 2020, and the pandemic period as April 2020 to December 2021 in all countries except for China, where the pandemic was declared by the end of January 2020.

The representativeness of the data regarding primary care physician visits varied by country (Supplementary Table S1). Further detailed information about the INTRePID datasets can be found elsewhere, as described in prior publications (16, 17).

### 2.3 Primary outcome

The primary outcome was monthly visits for respiratory conditions across different countries, taking into account both virtual and in-person visits and categorizing them based on the type of respiratory condition, regardless of age, gender or other demographic factors. Virtual visits included video-calls and telephone consultations between patients and primary care physicians. Consultations associated with diagnostic codes for respiratory conditions were identified in each country and divided into eight groups: asthma, emphysema/chronic obstructive pulmonary disease (COPD), respiratory allergies, other respiratory diseases, lower respiratory tract infection (LRTI), upper respiratory tract infection (URTI), influenza and COVID-19. Because of variations in reporting, we use the general term influenza to include all reported influenza-like illnesses. COVID-19 includes both suspected and confirmed cases as some coding systems do not differentiate between the two. These groups were also combined to form two major categories, chronic respiratory diseases (asthma, COPD, respiratory allergies, other respiratory diseases) and infectious respiratory diseases (LRTI, URTI, influenza, COVID-19). Singapore data specific to COVID-19 were unavailable in the first year of the pandemic because they were recorded in a different system. See Supplementary Tables S2–S10 for a full description of diagnostic codes and description of the billing coding systems used for categorization in each of the countries.

### 2.4 Statistical analysis

We conducted an analysis to compare pre-pandemic and pandemic time periods to determine the impact of COVID-19 on primary care for each country. We calculated the difference between the average volume of respiratory monthly visits before and after the onset of the COVID-19 pandemic in each country, along with 95% confidence intervals (CI) and associated *p*-values using Welch’s t-test, with a significance level of 0.05. This statistical approach was chosen to account for potential variations in sample sizes and variances between the two time periods. Additionally, we computed the percent change in average monthly visits from pre-pandemic to pandemic, along with 95% CI, for all countries and conditions.

Accounting for the variation within the different studied groups, we calculated the standardized difference in means using Cohen’s d test with Hedges correction. This adjustment accounts for biases in small sample sizes. Cohen’s d is defined as the difference in means divided by an estimate of the pooled standard deviation and incorporates a correction factor based on the size of the samples being compared (18, 19).

Furthermore, we visually presented the proportion of respiratory visits as a percentage of total visits by respiratory condition and by modality (in-person vs. virtual). These visit rates were calculated using total monthly visits (of any reason) as denominators except in Sweden and Peru. For these countries, we used total coded visits as the denominator, as uncoded visits were more likely to be with other health-care providers and potentially occurred concomitantly with a visit to a primary care physician. We generated a plot illustrating the trends in respiratory visits throughout the studied timeframe, alongside a trendline representing the incidence of new COVID-19 cases per 100,000 population. The data points for this plot were extracted from https://ourworldindata.org/coronavirus (20).

We supplemented our data analysis by conducting a survey among INTRePID collaborators, who served as points of contact in each country and were actively engaged in their local, regional, and national COVID response efforts. The survey aimed to describe the accessibility of care for patients with COVID-19 during the first and second years of the pandemic. Based on the framework developed by Huston et al. (6), which delineates the roles of various healthcare sectors in COVID-19 assessment, our survey employed a 5-point Likert scale offering respondents a range of options to indicate the frequency of occurrence for specific scenarios or activities related to COVID-19 care accessibility. Respondents could select from the following response options: “always,” “common,” “sometimes,” “occasionally,” and “never..” Refer to Supplementary Table S11 for a comprehensive description of the questionnaire employed.

### 2.5 Patient and public involvement

Patients and the public were not involved in the study design phase due to its highly technical nature; however, members of the public in INTRePID countries read the manuscript to ensure acceptable methods and interpretation. Specifically, the Patient and Clinician Engagement (PaCE) group, a well-established international patient advisory committee within the North American Primary Care Research Group (NAPCRG) (21), confirmed that our study was of public interest and offered important feedback on our results and discussion.

## 3 Results

**Figure 1:**
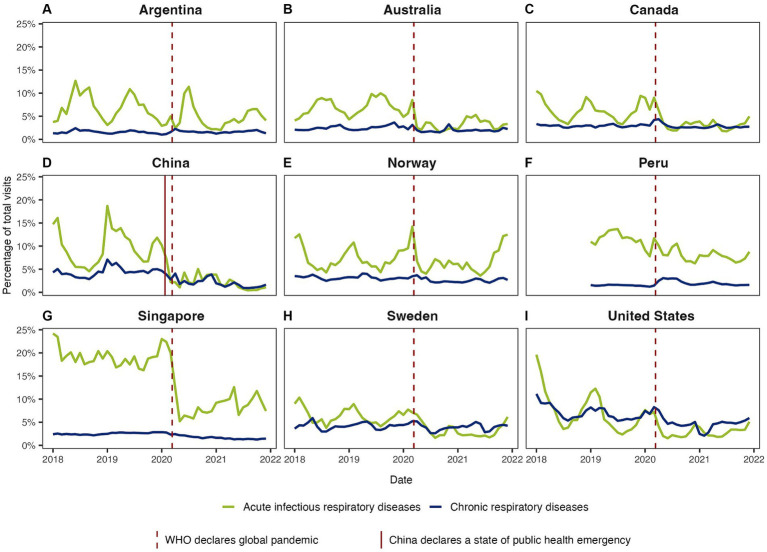
Overall visit rates for infectious and chronic respiratory illness during the pre-pandemic and pandemic periods in **(A)** Argentina, **(B)** Australia, **(C)** Canada, **(D)** China, **(E)** Norway, **(F)** Peru, **(G)** Singapore, **(H)** Sweden, and **(I)** United States. Trendlines show the monthly rates of respiratory visits over time. The rates were calculated by dividing the number of respiratory visits by the total visits to primary care services within each month and are expressed as percentages (%).

**Figure 2:**
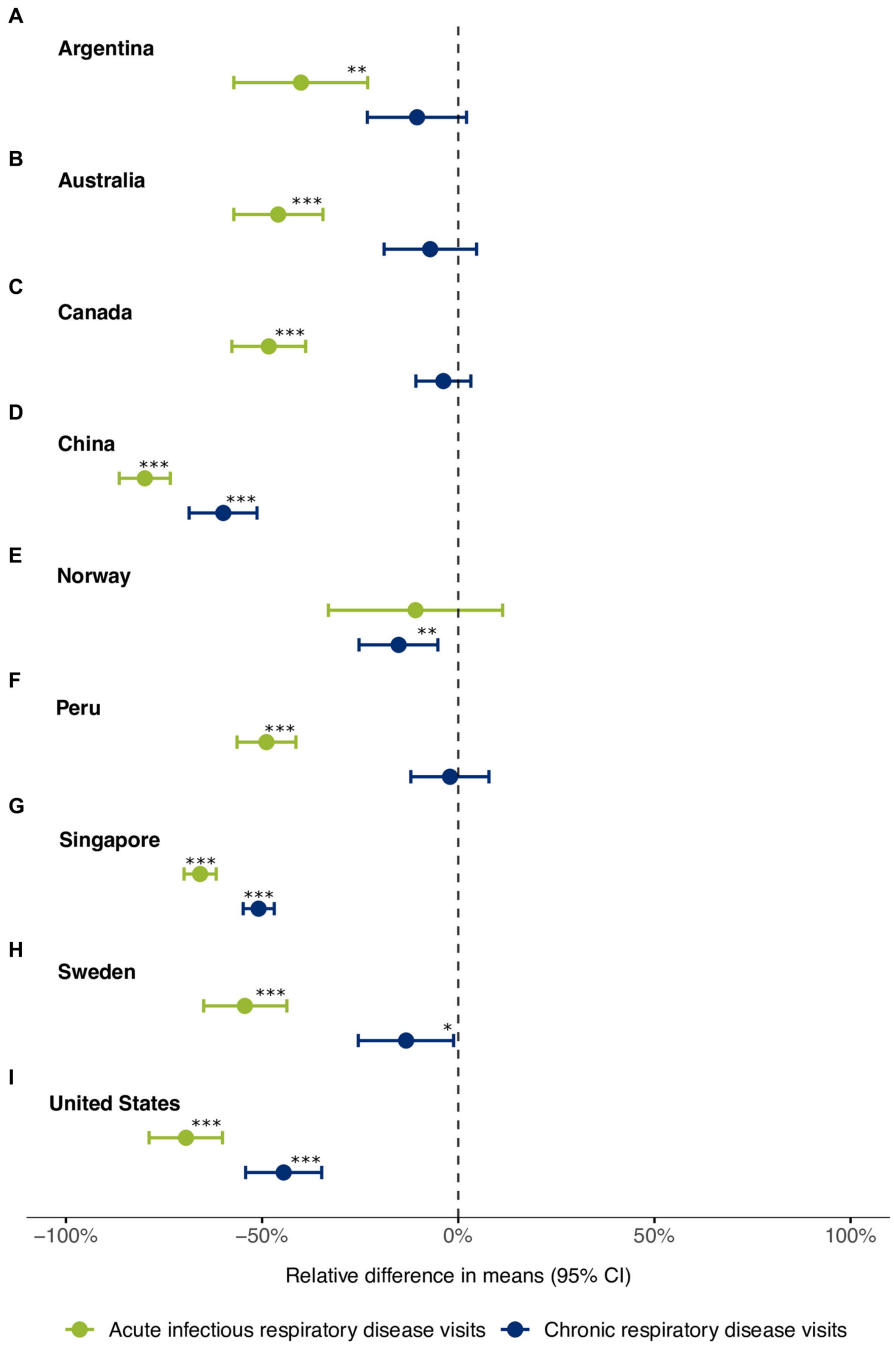
Relative difference in mean monthly visits for acute and chronic respiratory conditions between the pre-pandemic and the pandemic periods in **(A)** Argentina, **(B)** Australia, **(C)** Canada, **(D)** China, **(E)** Norway, **(F)** Peru, **(G)** Singapore, **(H)** Sweden, and **(I)** United States. Differences in mean monthly visits, 95% confidence intervals, and *p*-values were calculated using Welch’s t-test. The dots represent the relative difference in means between the two periods and are expressed as percentages (%). The error bars denote the 95% confidence intervals. Significance levels are indicated as follows: *p < 0.05; **p < 0.01; ***p < 0.001.

**Figure 3:**
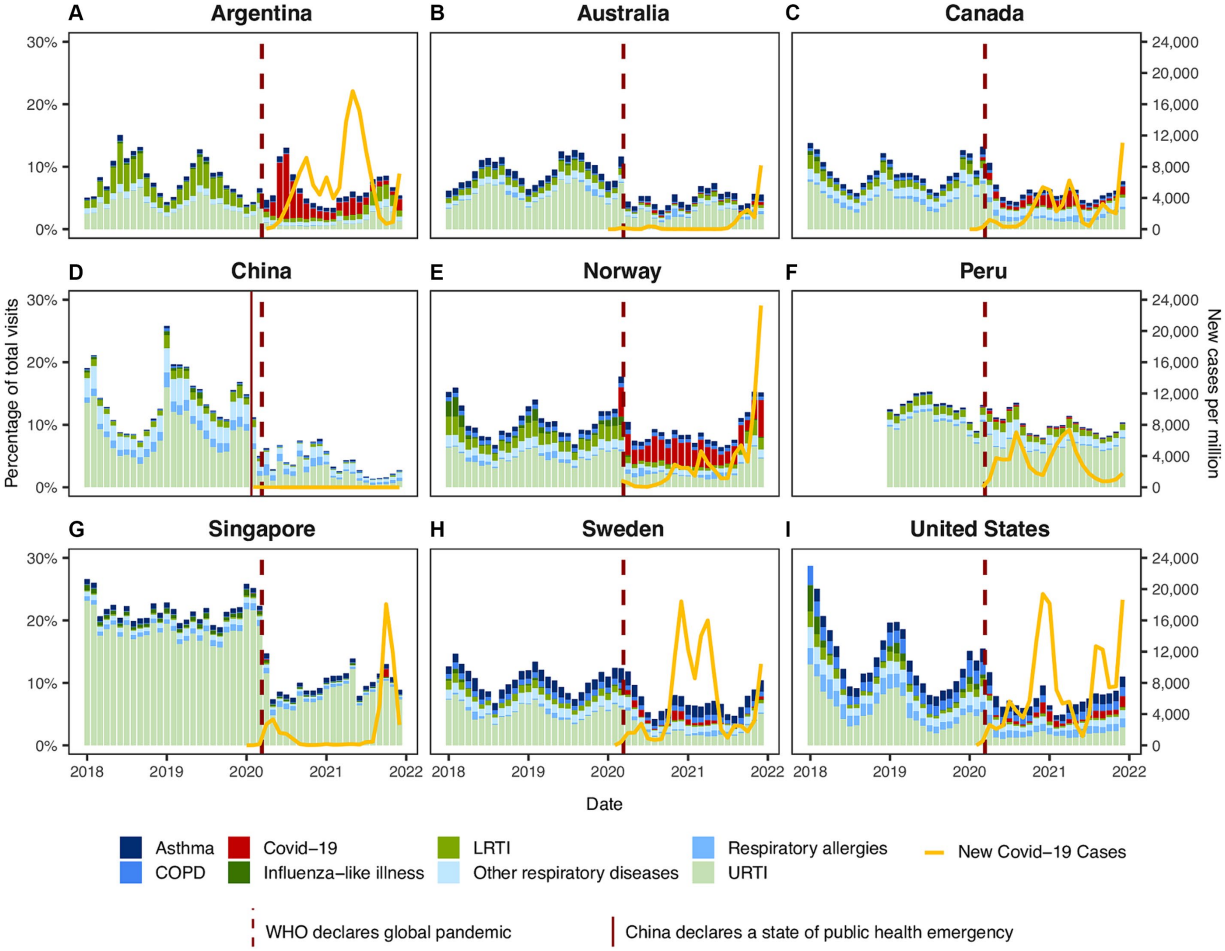
Respiratory visit rates by respiratory conditions in **(A)** Argentina, **(B)** Australia, **(C)** Canada, **(D)** China, **(E)** Norway, **(F)** Peru, **(G)** Singapore, **(H)** Sweden, and **(I)** United States. Stack column charts display monthly rates for different categories of respiratory visits. The rates were calculated by dividing the number of respiratory visits by the total visits to primary care services within each month and are expressed as percentages (%). The yellow line represents number of new COVID-19 cases per million. Source: https://ourworldindata.org/coronavirus (20).

For all INTRePID countries, when comparing the pandemic period with the pre-pandemic period, there was a decrease in infectious disease visits that was greater than the decrease observed for chronic respiratory illness visits (Figures 1, 2; Table 1). Decreases in the average number of monthly visits for acute infections ranged from −10.9% in Norway to −79.9% in China and were statistically significant in all countries (p = <0.05) except Norway. In Argentina and Norway, the reduction in acute respiratory infections was less pronounced because COVID-19 consultations contributed to almost half of these visits (Figure 3; Supplementary Figure S1). Decreases in chronic respiratory illness visits ranged from −2.1% to −59.9% and showed a statistically significant drop in China, Norway, Singapore, and the United States (Table 1; Figure 2). There was a drop in mean visit rates in all countries for most acute infectious respiratory conditions, with Singapore and the USA showing substantial declines in all sub-categories (Table 2; Supplementary Figure S2). Statistically significant changes in the average number of visits between the pandemic and pre-pandemic periods coincided with high standardized mean differences (exceeding 0.8 standard deviation units) (Tables 1, 2). It was interesting to note that in countries such as Canada, Norway, Peru, Sweden and in the US the patterns of respiratory condition visits in our primary care setting grossly mimicked the national COVID-19 waves (Figure 3).

**Table 1:** Change in average monthly acute and chronic respiratory visits in the pre-pandemic and pandemic periods.

				Pre-pandemic	Pandemic	Change in monthly mean visits between pandemic and pre-pandemic periods
Country	Mean (SD)	Mean (SD)	Absolute mean change (95% CI)^1^	*P*-value^1^	Relative mean change % (95% CI)	Standardized mean difference (95% CI)^2^
Argentina						
Acute infectious respiratory disease visits	6,770 (3,238)	4,054 (2,094)	-2,717 (−4,274, −1,160)	**0.001**	−40.1 (−57.2, −23.1)	**−0.95 (−1.56, −0.34)**
Chronic respiratory disease visits	1,549 (436)	1,386 (303)	−163 (−378, 52)	0.13	−10.5 (−23.2, 2.1)	−0.4 (−1.00, 0.16)
Australia						
Acute infectious respiratory disease visits	8,866 (2,936)	4,800 (1,891)	−4,066 (−5,476, −2,656)	**<0.001**	−45.9 (−57.2, −34.5)	**−1.58 (−2.24, −0.92)**
Chronic respiratory disease visits	3,184 (802)	2,956 (587)	−227 (−631, 176)	0.5	−7.16 (−18.9, 4.67)	−0.31 (−0.89, 0.27)
Canada						
Acute infectious respiratory disease visits	4,459 (1,516)	2,306 (693)	−2,153 (−2,818, −1,488)	**<0.001**	−48.3 (−57.7, −38.9)	**−1.72 (−2.40, −1.05)**
Chronic respiratory disease disease visits	2,107 (285)	2,026 (246)	−81 -235, (74)	0.3	−3.8 (−10.8, 3.2)	0.29 (−0.87, 0.28)
China						
Acute infectious respiratory disease visits	672 (272)	135 (93)	−537 (−655, −419)	**<0.001**	−79.9 (−86.4, −73.4)	**−2.55 (−3.32, −1.78)**
Chronic respiratory disease visits	307 (71)	123 (59)	−184 (−222, −146)	**<0.001**	−59.9 (−68.6, −51.3)	**−2.75 (−3.54, −1.95)**
Norway						
Acute infectious respiratory disease visits	93,239 (35,920)	83,094 (39,330)	−10,145 (−32,400, 12,110)	0.4	−10.9 (−33.1, 11.3)	−0.26 (−0.84, 0.31)
Chronic respiratory disease visits	38,431 (6,492)	32,572 (7,591)	−5,858 (−10,054, −1,663)	**0.007**	−15.2 (−25.3, −5.2)	**−0.82 (−1.42–0.22)**
Peru						
Acute infectious respiratory disease visits	497,476 (82,097)	254,339 (71,894)	−243,136 (−297,181, −189,092)	**<0.001**	−48.9 (−56.4, −41.4)	**−3.11 (−4.11–2.12)**
Chronic respiratory disease visits	64,405 (7,975)	63,022 (11,738)	−1,383 (−8,062, 5,296)	0.7	−2.1 (−12.1, 7.8)	−0.13 (−0.80, 0.54)
Singapore						
Acute infectious respiratory disease visits	19,815 (2,473)	6,776 (1,732)	−13,039 (−14,263, −11,816)	**<0.001**	−65.8 (−69.9, −61.7)	**−5.88 (−7.12, −4.56)**
Chronic respiratory disease visits	2,612 (284)	1,283 (209)	−1,329 (−1,472, −1,185)	**<0.001**	−50.9 (−54.8, −46.9)	**−5.14 (−6.33, −3.95)**
Sweden						
Acute infectious respiratory disease visits	2,069 (584)	944 (451)	−1,125 (−1,426, −824)	**<0.001**	−54.4 (−64.9, −43.7)	**−2.09 (−2.80, −1.37)**
Chronic respiratory disease visits	1,365 (315)	1,183 (305)	−182 (−364, −0.69)	**0.049**	−13.3 (−25.5, −1.2)	−0.58 (−1.16, 0.01)
United States						
Acute infectious respiratory disease visits	1,375 (880)	420 (183)	−954 (−1,310, −599)	**<0.001**	−69.4 (−78.8, −60.1)	**−1.40 (−2.04, −0.75)**
Chronic respiratory disease visits	1,321 (350)	733 (246)	−588 (−761, −414)	**<0.001**	−44.5 (−54.2, −34.8)	**−1.86 (−2.56, −1.18)**

^1^Welch Two Sample t-test. ^2^Hedges’g effect size statistics. The magnitude is assessed using the thresholds provided by Cohen (22): <(+/−)0.2 “negligible,” <(+/−)0.5 “small,” <(+/−)0.8 “medium,” (+/−) > 0.8 “large”. Bolded values denote statistical significance at the 95% confidence level or a standardized mean difference of large magnitude.

**Table 2:** Change in average monthly respiratory visits by category in the pre-pandemic and pandemic periods.

	Pre-pandemic	Pandemic	Change in monthly mean visits between pandemic and pre-pandemic periods			
Country	Mean (SD)	Mean (SD)	Absolute mean change (95% CI)^1^	*P*-value^1^	Relative mean change % (95% CI)	Standardized mean difference (95% CI)^2^
Argentina						
URTI	3,830 (1,601)	1,090 (984)	−2,740 (−3,496, −1,983)	**<0.001**	−71.5 (−83.4, −59.7)	**−1.97 (−2.67, −1.27)**
LRTI	2,757 (1,560)	858 (661)	−1,898 (−2,573, −1,224)	**<0.001**	−68.9 (−81.1, −56.7)	**−1.49 (−2.14, −0.84)**
Influenza-like illness	184 (150)	15 (17)	−169 (−228, −109)	**<0.001**	−91.8 (−96.5, −87.2)	**−1.46 (−2.10, −0.81)**
Asthma	549 (183)	646 (165)	98 (−3.9, 199)	0.059	17.7 (−1.9, 37.3)	0.55 (−0.04, 1.13)
COPD	106 (24)	92 (17)	−14 (−26, −2.4)	**0.019**	−13.2 (−23.3, −3.1)	−0.67 (−1.26, −0.07)
Respiratory allergies	55 (22)	53 (18)	−1.9 (−14, 10)	0.7	−3.6 (−23.8, 16.6)	−0.09 (−0.66, 0.48)
Other respiratory diseases	839 (245)	595 (205)	−245 (−375, −114)	**<0.001**	−29.1 (−42.1, −16)	**−1.05 (−1.67, −0.44)**
Australia						
URTI	7,257 (2,379)	3,846 (1,619)	−3,411 (−4,575, −2,246)	**<0.001**	−47.0 (−58.6, −35.4)	**−1.61 (−2.27, −0.95)**
LRTI	1,490 (501)	608 (311)	−882 (−1,120, −645)	**<0.001**	−59.2 (−69.5, −48.9)	**−2.02 (−2.73, −1.32)**
Influenza-like illness	119 (112)	42 (64)	−76 (−128, −25)	**0.005**	−64.7 (−90.9, −38.5)	**−0.80 (−1.40, −0.20)**
Asthma	1,387 (297)	1,340 (211)	−47 (−195, 101)	0.5	−3.4 (−13.6, 6.8)	−0.17 (−0.75, 0.40)
COPD	295 (55)	293 (37)	−1.6 (−28, 25)	>0.9	−0.7 (−9.5, 8.1)	−0.03 (−0.61, 0.54)
Respiratory allergies	349 (369)	479 (480)	130 (−126, 387)	0.3	37.2 (−43.1, 117.6)	0.30 (−0.27, 0.88)
Other respiratory diseases	1,153 (325)	844 (247)	−309 (−475, −143)	**<0.001**	−26.8 (−38.8, −14.8)	**−1.03 (−1.64, −0.42)**
Canada						
URTI	3,481 (1,082)	1,282 (500)	−2199 (−2,674, −1,723)	**<0.001**	−63.2 (−70.7, −55.7)	**−2.46 (−3.22, −1.70)**
LRTI	824 (309)	194 (79)	−629 (−755, −503)	**<0.001**	−76.5 (−81.7, −71.2)	**−2.60 (−3.38, −1.82)**
Influenza-like illness	142 (149)	30 (29)	−114 (−174, −53)	**<0.001**	−78.9 (−91, −66.8)	**−0.98 (−1.59, −0.37)**
Asthma	504 (93)	437 (73)	−67 (−115, −19)	**0.007**	−13.3 (−21.9, −4.7)	−0.78 (−1.37, −0.18)
COPD	277 (40)	205 (32)	−72 (−93, −51)	**<0.001**	−26 (−32.4, −19.6)	**−1.93 (−2.62, −1.24)**
Respiratory allergies	364 (126)	457 (149)	93 (11, 175)	**0.028**	25.5 (1.6, 49.5)	0.67 (0.08, 1.26)
Other respiratory diseases	962 (176)	928 (125)	−35 (−122, 53)	0.4	−3.5 (−12.2, 5.1)	−0.21 (−0.79, 0.36)
China						
URTI	586 (241)	124 (89)	−461 (−567, −356)	**<0.001**	−78.8 (−85.9, −71.8)	**−2.46 (−3.22, −1.70)**
LRTI	72 (29)	11 (8)	−61 (−74, −49)	**<0.001**	−84.7 (−89.9, −79.6)	**2.76 (−3.56, −1.96)**
Influenza-like illness	14 (17)	<1	−14 (−21, −6.9)	-	-	**-**
Asthma	11.9 (5.2)	9.0 (6.3)	−3 (−6.3, 0.40)	0.083	−24.4 (−49.6, 0.9)	−0.50 (−1.09, 0.07)
COPD	12.8 (5.8)	12.4 (6.7)	−0.33 (−4.0, 3.4)	0.9	−3.1 (−30.6, 24.3)	−0.50 (−0.62, 0.52)
Respiratory allergies	92 (25)	42 (24)	−50 (−64, −36)	**<0.001**	−54.3 (−66.1, −42.6)	**−2.00 (−2.70, −1.30)**
Other respiratory diseases	189 (48)	59 (30)	−130 (−153, −107)	**<0.001**	−68.8 (−76, −61.6)	**−3.14 (−4.0, −2.29)**
Norway						
URTI	58,628 (15,284)	31,607 (22,865)	−27,021 (−38,802, −15,240)	**<0.001**	−46.1 (−63.6, −28.6)	**−1.40 (−2.04, −0.76)**
LRTI	22,937 (7,141)	10,894 (8,470)	−12,043 (−16,701, −7,384)	**<0.001**	−52.5 (−69.3, −35.8)	**−1.52 (−2.18, −0.87)**
Influenza-like illness	9,302 (10,003)	1,214 (848)	−8,088 (−12,060, −4,116)	**<0.001**	−86.9 (−93.5, −80.4)	**−1.05 (−1.67, −0.44)**
Asthma	9,316 (1,880)	8,697 (2,181)	−620 (−1,829, 589)	0.3	−6.6 (−18.9, 5.6)	−0.30 (−0.88, 0.27)
COPD	8,272 (1,015)	7,034 (1,204)	−1,238 (−1,900, −576)	**<0.001**	−15 (−22.3, −7.6)	**−1.10 (−1.72, −0.49)**
Respiratory allergies	4,881 (3,793)	5,846 (4,331)	964 (−1,449, 3,378)	0.4	19.8 (−31.9, 71.5)	0.23 (−0.34, 0.81)
Other respiratory diseases	15,961 (3,421)	10,996 (4,873)	−4,965 (−7,506, −2,424)	**<0.001**	−31.1 (−45.3, −16.9)	**−1.18 (−1.81, −0.56)**
Peru						
URTI	443,498 (72,828)	214,486 (67,005)	−229,012 (−277,751, −180,273)	**<0.001**	−51.6 (−59.3, −44.0)	**−3.22 (−4.23, −2.21)**
LRTI	42,873 (8,372)	29,356 (8,702)	−19,310 (−25,955, −12,665)	**<0.001**	−31.5 (−42.5, −20.5)	**−1.97 (−2.78, −1.15)**
Influenza-like illness	495 (61)	608 (481)	112 (−108, 333)	0.3	22.8 (−19.4, 65.1)	−0.30 (−0.38, 0.97)
Asthma	13,561 (2,590)	6,154 (1,353)	−7,407 (−8,934, −5,879)	**<0.001**	−54.6 (−60.7, −48.5)	**−3.69 (−4.80, −2.60)**
COPD	4,601 (862)	1,635 (401)	−2,965 (−3,467, −2,464)	**<0.001**	−64.5 (−69.5, −59.4)	**−4.58 (−5.84, −3.32)**
Respiratory allergies	15,423 (3,321)	7,454 (2,698)	−7,969 (−10,106, −5,832)	**<0.001**	−51.7 (−60.8, −42.5)	**−2.62 (−3.53, −1.71)**
Other respiratory diseases	30,821 (2,317)	47,779 (8,871)	16,958 (12,773, 21,142)	**<0.001**	55 (41.4, 68.7)	2.38 (1.50, 3.25)
Singapore						
URTI	18,985 (2,323)	6,527 (1,661)	−12,459 (−13,617, −11,300)	**<0.001**	−65.6 (−69.7, −61.6)	**−5.94 (−7.27, −4.61)**
LRTI	203 (29)	60 (34)	−143 (−162, −124)	**<0.001**	−70.4 (−77.8, −63.1)	**−4.46 (−5.53, −3.39)**
Influenza-like illness	624 (171)	135 (66)	−489 (−562, −416)	**<0.001**	−78.4 (−83.4, −73.3)	**−3.55 (−4.46, −2.63)**
Asthma	813 (70)	579 (58)	−234 (−271, −197)	**<0.001**	−28.8 (−32.6, −25.0)	**−3.55 (−4.47, −2.63)**
COPD	108 (15)	73 (10)	−35 (−42, −27)	**<0.001**	−32.4 (−37.7, −27.1)	**−2.62 (−3.40, −1.84)**
Respiratory allergies	852 (144)	447 (145)	−405 (−490, −320)	**<0.001**	−47.5(−55.6, −39.5)	**−2.76 (−3.56, −1.96)**
Other respiratory diseases	839 (181)	184 (72)	−655 (−732, −577)	**<0.001**	−78.1 (−82.2, −74.0)	**−4.47 (−5.54, −3.40)**
Sweden						
URTI	1,715 (462)	722 (381)	−993 (−1,237, −748)	**<0.001**	−57.9 (−68.3, −47.5)	**−2.28 (−3.02, −1.54)**
LRTI	314 (85)	69 (38)	−245 (−282, −208)	**<0.001**	−78.0 (−83.7, −72.4)	**−3.51 (−4.43,-2.60)**
Influenza-like illness	40 (56)	3 (7)	−37 (−59, −14)	**0.002**	−92.5 (−101, −84.0)	**−0.85 (−1.45, −0.25)**
Asthma	506 (135)	507 (152)	1.4 (−84, 86)	>0.9	0.2 (−16.1, 16.5)	0.00 (−0.57, 0.58)
COPD	282 (69)	233 (72)	−49 (−91, −7.8)	**0.021**	−17.4 (−30.7, −4.1)	−0.69 (−1.28, −0.09)
Respiratory allergies	111 (91)	123 (75)	12 (−37, 60)	0.6	10.8 (−34.0, 55.6)	0.14 (−0.44, 0.71)
Other respiratory diseases	466 (88)	320 (85)	−146 (−197, −95)	**<0.001**	−31.3 (−40.5, −22.1)	**−1.65 (−2.32, −0.98)**
United States						
URTI	1,053 (593)	263 (107)	−790 (−1,029, −552)	**<0.001**	−75.0 (−81.9, −68.2)	**−1.72 (−2.39, −1.04)**
LRTI	202 (113)	48 (18)	−154 (−199, −109)	**<0.001**	−76.2 (−82.5, −69.9)	**−1.77 (−2.45, −1.09)**
Influenza-like illness	120 (203)	3 (4)	−117 (−198, −37)	**0.006**	−97.5 (−99.6, −95.4)	−0.75 (−1.35–0.16)
Asthma	302 (46)	239 (78)	−63 (−102, −24)	**0.003**	−20.9 (−32.8, −8.9)	**−0.99 (−1.60, −0.38)**
COPD	346 (101)	161 (64)	−184 (−233, −136)	**<0.001**	−53.5 (−63.7, −43.3)	**−2.08 (−2.80, −1.37)**
Respiratory allergies	340 (132)	178 (54)	−162 (−219, −105)	**<0.001**	−47.6 (−57.9, −37.4)	**−1.52 (−2.17, −0.86)**
Other respiratory diseases	333 (118)	155 (63)	−178 (−232, −124)	**<0.001**	−53.5 (−63.7, −43.3)	**−1.78 (−2.46, −1.10)**

^1^Welch Two Sample *t*-test. ^2^Hedges’g effect size statistics. The magnitude is assessed using the thresholds provided by Cohen (22): <(+/−)0.2 “negligible,” <(+/−)0.5 “small,” <(+/−)0.8 “medium,” (+/−) > 0.8 “large”. Bolded values denote statistical significance at the 95% confidence level or a standardized mean difference of large magnitude.

Most of the INTRePID countries reported less seasonal variation for infectious respiratory disease during the pandemic compared to the pre-pandemic period. Supplementary Figures S3–S10 show monthly rates of respiratory visits by category in each country. These data demonstrated no influenza spike during the first 2 years of the pandemic (Supplementary Figure S3). Similar to influenza, visits for other URTI declined across INTRePID countries (Supplementary Figure S4). As expected, with a decline in influenza, LRTIs, including pneumonia, decreased dramatically during the pandemic (Supplementary Figure S5).

Primary care visits for COVID-19 varied between the INTRePID countries with different fluctuations over time. Australia, China, Singapore, and Peru had few COVID-19 visits in primary care (0–1.3% of total visits). Conversely, Argentina and Norway reported large numbers of primary care COVID-19 visits accounting for 5.9–10.4% of visits. Canada, Sweden, and the United States had a moderate rate of COVID-19 visits accounting for 1.6–2.6% of all visits (Figure 3; Supplementary Figures S1, S6).

Seasonal variation for respiratory allergies continued throughout the pandemic (Supplementary Figure S7). COPD visits were fairly constant during the pandemic with slight variation. Norway, Canada, and the United States reported a modest decline in COPD visits, while the other countries were essentially unchanged (Supplementary Figure S8). Asthma visit rates showed little change during the pandemic (Supplementary Figure S9).

**Figure 4:**
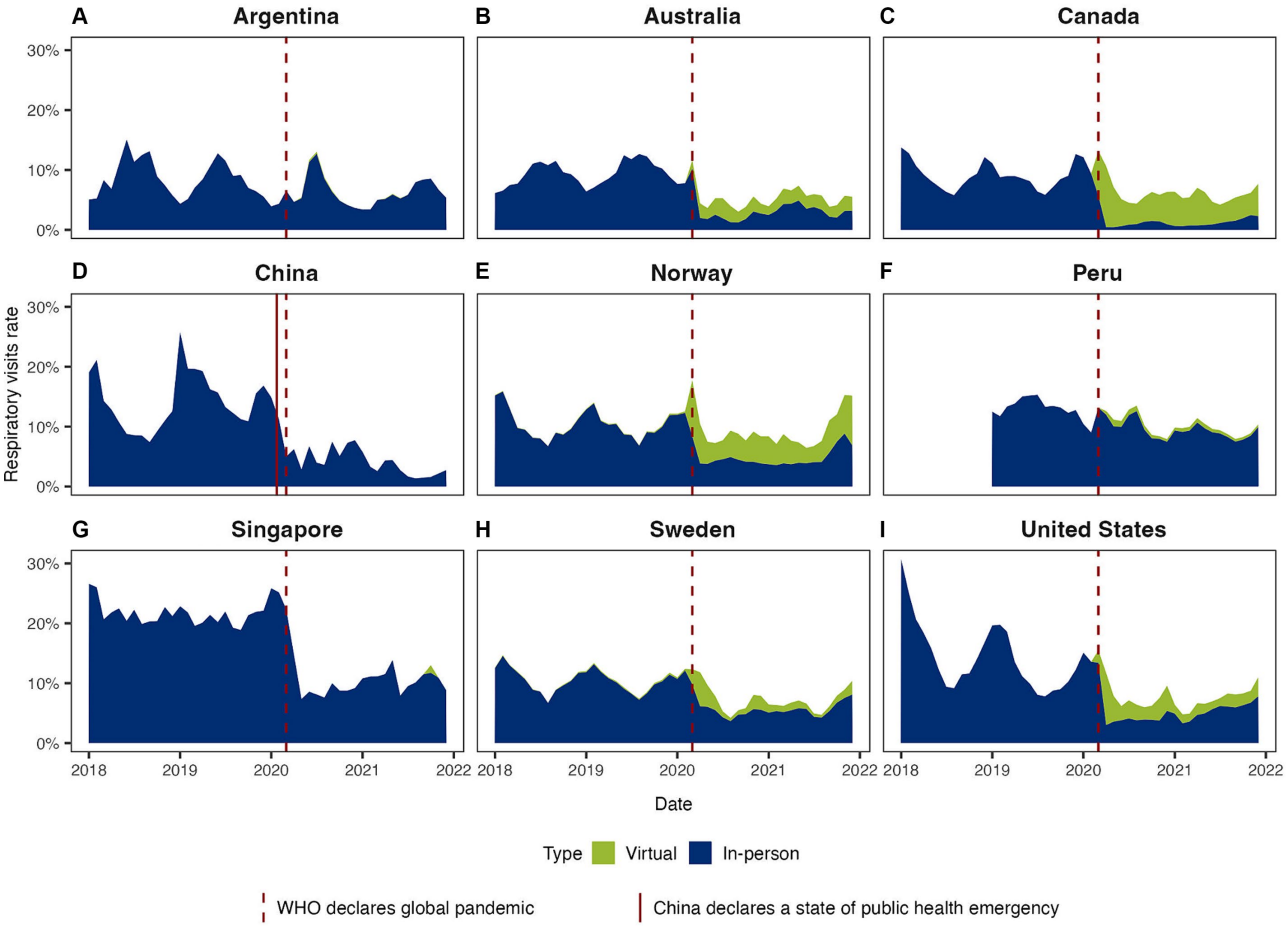
Respiratory visit rates by visit modality (virtual vs. in-person) in **(A)** Argentina, **(B)** Australia, **(C)** Canada, **(D)** China, **(E)** Norway, **(F)** Peru, **(G)** Singapore, **(H)** Sweden, and **(I)** United States. Area graphs illustrate respiratory visit rates by modality of care. The rates were calculated by dividing the number of respiratory visits by the total visits to primary care services within each month and are expressed as percentages (%).

Virtual visits for respiratory conditions in Canada were more common than in-person visits. In Norway, virtual visit rates were comparable to in-person visit rates, while in other countries, virtual visit rates were either negligible or lower than in-person visits during the pandemic (Figure 4).

The survey conducted among INTRePID representatives from participating countries revealed that assessment centers were the most frequent sites for COVID-19 diagnosis in both the first and second years of the pandemic (Table 3). Many countries mentioned emergency departments as sites for COVID-19 diagnosis, although their prevalence slightly decreased during the second year of the pandemic. Primary care settings occasionally served as sites for COVID-19 assessment, with a slight increase noted during 2021. In contrast, virtual visits were prevalent throughout the first two years of the pandemic. Table 3 summarizes the survey responses.

**Table 3:** Site of assessment of potential COVID patients among INTRePID countries.

Country	Virtual	Primary care offices	Assessment centers	After-hours clinics	Emergency departments	Home visits
Argentina						
2020 2021	Common Common	Sometimes Sometimes	Common Common	Sometimes Occasional	Common Common	Sometimes Common
Australia						
2020 2021	Common Common	Occasional Occasional	Common Common	Sometimes Sometimes	Sometimes Sometimes	Occasional Occasional
Canada						
2020 2021	Sometimes Sometimes	Sometimes Sometimes	Common Common	Sometimes Sometimes	Common Common	Occasional Occasional
China						
2020 2021	Never Never	Occasional Sometimes	Common Common	Sometimes Sometimes	Common Common	Never Never
Norway						
2020 2021	Common Common	Sometimes Sometimes	Common Common	Common Common	Occasional Occasional	Occasional Occasional
Peru						
2020 2021	Sometimes Sometimes	Occasional Sometimes	Common Common	Never Never	Common Common	Sometimes Occasional
Singapore						
2020 2021	Never Sometimes	Common Common	Common Sometimes	Common Common	Common Sometimes	Never Never
Sweden						
2020 2021	Common Common	Sometimes Occasional	Common Common	Sometimes Sometimes	Common Common	Occasional Occasional
United States						
2020 2021	Common Sometimes	Occasional Common	Common Sometimes	Occasional Sometimes	Sometimes Occasional	Occasional Occasional

## 4 Discussion

### 4.1 Impact of COVID-19 pandemic on primary care visits for respiratory illnesses

Our study found a notable decline in the rate and average monthly volume of primary care visits for respiratory concerns following the onset of the COVID-19 pandemic. Acute respiratory infection visits experienced a more pronounced decrease compared to non-infectious chronic respiratory illness visits. This trend suggests that COVID-19 mitigation measures likely impacted infections such as influenza and URTIs, while chronic respiratory illnesses such as COPD and asthma were less amenable to these efforts. Predictably, COVID-19 had a lesser effect on seasonal respiratory illnesses such as allergies. Our findings align with existing research demonstrating a decrease in respiratory virus activity during the COVID-19 pandemic. This decline has been linked to reductions in cases of acute respiratory illnesses and influenza-like illnesses. Furthermore, studies have shown a positive impact on chronic respiratory diseases, with fewer hospital admissions for asthma and COPD exacerbations during the pandemic’s early stages, which coincides with the implementation of national lockdowns and non-pharmaceutical interventions (23).

The changes observed in non-infectious respiratory illnesses might stem from reduced access to in-person primary care or insufficient reporting of virtual visits not accounted for in the available data. Before COVID-19, only a few primary care practices conducted virtual visits, and there may have been delays in recording, coding, and reporting virtual visits during the early phase of the pandemic.

### 4.2 Regional variations in respiratory illness visits

While non-COVID-19 acute infection visits generally decreased, this change varied across countries participating in INTRePID. For example, respiratory illness accounted for 21.8% of visits in Singapore before the onset of the COVID-19 pandemic. This rate dropped below 10% of visits in the first few months of the pandemic and slowly levelled out at just over 10% of visits, with very few attributed to COVID-19. Prior to COVID-19, there was seasonal variation in Canada, with peaks near 15% of primary care visits attributed to respiratory illness. While there was an overall decrease in the rate of respiratory illness, it still hovered around 5%, with a significant number of COVID-19 patients. Our results were similar to those found in the United Kingdom, showing a marked decline in acute respiratory illness with flattening of seasonal variation while maintaining the usual incidence and seasonal variation associated with allergic rhinitis (24).

The variation in respiratory illness visits across countries also highlights differences in the location of COVID-19 infection care within our study population. Reflecting the diverse healthcare landscapes and pandemic responses globally, the dominant sites of COVID-19 care varied significantly. These variations encompass the dominant sites of COVID-19 care, including primary care versus other facilities, and align with the fluctuating waves of COVID-19 infections across the globe. Huston et al. studied the primary care and public health response to COVID-19 in 6 countries in early 2020 at the start of the pandemic (6). They found that COVID-19 assessment centers were the dominant location for triage of potential COVID-19 cases. In accordance with the Houston et al. study, neither their analysis nor our study identified primary care practices as the predominant COVID-19 testing and assessment locations in Australia, Canada, or the United States. Devi and colleagues reported that the majority of patients in their multi-country study had seen a health professional during the pandemic (63%) (25). This was even higher in Argentina, the only country that overlaps our research, which may explain the smaller drop in overall visit volume seen in this country.

### 4.3 Impact of COVID-19 pandemic on infectious respiratory illnesses

There is evidence that many infectious respiratory illnesses were much less common during the first 1–2 years of the COVID-19 pandemic (26, 27). Stephenson reported that while overall ambulatory visits dropped by just 5% between 2019 and 2020, the number of “common cold” visits dropped by 51% (3). Rodgers et al. reported that during the first few months of the pandemic, respiratory visits to the emergency department (ED) were twice the pre-pandemic rate; however, by the end of 2020, ED respiratory infections were below pre-pandemic rates (28). Liu et al. found lower rates of most respiratory pathogens among hospitalized children with lower respiratory tract infections (29). Lockdowns, social distancing, and mask mandates may have contributed to protection from COVID-19 (30, 31) and many other endemic and seasonal infections such as respiratory syncytial virus (RSV), influenza and other common rhino and adenoviruses (32, 33). The lower rate of respiratory infections seen among INTRePID participants may indicate the response to robust public health measures aimed at minimizing the spread of contagious illness. With the reduction in COVID-19 mitigation efforts in 2021, there was a resurgence in common respiratory infections (34, 35). Most recent evidence from December 2022 in the United States and Norway revealed major increases in RSV and influenza (36). Renati and Linder reported that a majority of acute respiratory infections may not require a clinical consultation (37). The additional fear of transmission and the restrictions in place in many healthcare settings may have been enough to keep patients with mild to moderate COVID-19 infection away from the clinic. Bullen et al., in a 9-country survey, found that 60% of physicians and pharmacists reported patient “reluctance to visit a healthcare setting.” (38).

Insights gained from previous pandemics provide crucial context for understanding the dynamics of viral interactions during outbreaks. For instance, the emergence of influenza A (H1N1) pandemic in 2009 had a significant impact on the circulation of other respiratory viruses between 2009 and 2011 (39). Studies observed unusual patterns in virus activity following the influenza A (H1N1) pandemic peak. Research conducted in France suggested a delay in the circulation of respiratory syncytial virus (RSV) during the 2009–2010 season, compared to previous years (40). Similarly, a study conducted in the United Kingdom found that some cases initially diagnosed as influenza during the summer outbreak were actually caused by other respiratory viruses (41). These findings underscore the interplay between dominant strains like the influenza A (H1N1) during the 2009 pandemic and other respiratory viruses. While our study focused on acute respiratory visits without examining the specific viruses involved, similar dynamics were observed during the COVID-19 pandemic (39).

### 4.4 Role of primary care in pandemic response

Goodyear-Smith and colleagues found that the perceived strength of the primary care system was not associated with a lower COVID-19 mortality rate (42). However, they also found that the perceived strength of a pandemic plan with robust implementation was associated with lower COVID-19 mortality. Local, regional, and national planning for COVID-19 recovery should also include planning for the management and resurgence of other respiratory infections. Primary care plays a crucial role in vaccination and may need to be part of post-pandemic immunization catchup and annual management (43, 44). Virtual visits may also play an important role in primary care and require further research to maximize their impact.

### 4.5 Limitations

This research comes with some limitations. First, we collected data from numerous sources with large variations in availability. While some INTRePID countries provided comprehensive national-level data, others provided limited data from a few clinics or regions. For instance, data from Peru (45) include over 8000 primary care practices, representing nearly 70% of the population. Data from smaller samples, such as those from China and the US, may not reflect the regional variation nor the national experience in primary care. Also, the usage of country names to define the regions is primarily for clarity and comprehension purposes rather than a direct comparison between the entire country populations. However, we have at least in part achieved a global footprint in relation to the sampling frame.

Our aim was not necessarily to compare countries to each other but rather to compare pre-pandemic with COVID-19 pandemic time periods among the participating INTRePID countries. We present unadjusted analyses as social and demographic variables were not available among all participant data. As many countries managed COVID-19 outside of the typical primary care setting, the COVID-19-related visits presented here reflect the impact on and role of primary care in the typical primary care settings rather than the full impact of COVID-19 in the community.

Moreover, primary care physicians staffed COVID-19 assessment clinics established outside the conventional primary care settings in numerous INTRePID countries. However, the visit rates for these patients were not accounted for in our data, except in Norway.

Another limitation arises from the reliance on coding systems themselves. For instance, due to the emergence of COVID-19 as a new diagnosis, some coding systems did not differentiate between suspected and confirmed cases. Unfortunately, this limitation in data availability prevented us from making a stratified analysis of suspected and confirmed cases. However, given the widespread epidemic nature of COVID-19, in the midst of a local wave, it is likely that most suspected cases were true COVID-19 infections.

We acknowledge that COVID-19-related visits to primary care do not reflect COVID-19 cases or death rates (20). The results presented in this study do not represent the impact of the global COVID-19 pandemic on primary care. We show the impact of the pandemic within each of the participant countries and particular regions involved.

Telehealth virtual visits are a safe and effective alternative to in-person clinic visits (14). While it has limitations, such as the inability to perform physical exams (46), it allows for efficient triage, effective symptom assessment, and the provision of timely medical advice, especially during times when in-person visits were restricted. Many primary care practices increased their use of virtual telehealth visits throughout the COVID-19 pandemic, particularly in the first year of the pandemic. While the INTRePID data included in-person as well as virtual visits, the rapid shift to virtual visits may not have generated a full encounter in the medical record or billing number in some countries, resulting in a loss of primary care practice visit data. Virtual visits, particularly early in the pandemic, may have been audio only and did not generate a full encounter in some countries’ medical or billing records.

## 5 Conclusion

The COVID-19 pandemic resulted in a major impact on primary care visits and reasons for visits. As expected from widespread physical distancing and mask mandates, there was a decreased rate of respiratory illness presentation in primary care after the start of the pandemic. INTRePID countries exhibited substantial variations. Primary care in all countries continued to provide service, in-person and through virtual telehealth consultation, for respiratory conditions as well as other health needs. Primary care is pivotal in epidemic and pandemic infection management. Understanding the role of primary care may provide valuable information for COVID-19 recovery efforts and planning for future global pandemic emergencies.

### 5.1 Implications for future research

Future research should explore the long-term impact of the pandemic on primary care utilization patterns and healthcare delivery. Further investigation into the effectiveness of virtual visits and strategies to address the underreporting of encounters is warranted. Moreover, understanding the interplay between pandemic response measures and the resurgence of respiratory infections will inform future public health interventions and pandemic preparedness efforts. This study also identifies the urgent need to consider methods to harmonize and curate data from various sources as a method to conduct robust international primary care research.

## Data availability statement

The data analyzed in this study is subject to the following licenses/restrictions: All relevant data is contained within the article. The original contributions presented in the study are included in the article and supplementary files, further inquiries can be directed to the corresponding author. However, data sharing is governed by local regulations, which differ across countries. Individual-level data is not accessible to the public due to research ethics approval restrictions. Analytic code for data analysis is available upon request. Requests to access these datasets should be directed to k.tu@utoronto.ca.

## Ethics statement

The studies involving humans were approved by this study received Research Ethics Board approval from the University of Toronto #40943. The studies were conducted in accordance with the local legislation and institutional requirements. Written informed consent for participation was not required from the participants or the participants’ legal guardians/next of kin in accordance with the national legislation and institutional requirements.

## Author contributions

JW: Conceptualization, Investigation, Methodology, Visualization, Writing – original draft. AB: Conceptualization, Formal analysis, Investigation, Methodology, Writing – review & editing. ML: Conceptualization, Data curation, Formal analysis, Investigation, Methodology, Visualization, Writing – review & editing. PZ: Data curation, Investigation, Validation, Writing – review & editing. WW: Data curation, Investigation, Validation, Writing – review & editing. KW: Data curation, Investigation, Validation, Writing – review & editing. WP: Data curation, Investigation, Validation, Writing – review & editing. JS-V: Data curation, Investigation, Validation, Writing – review & editing. LS: Data curation, Investigation, Validation, Writing – original draft. AN: Data curation, Investigation, Validation, Writing – review & editing. J-AM-N: Data curation, Investigation, Validation, Writing – review & editing. ZJL: Data curation, Investigation, Validation, Writing – review & editing. ZL: Data curation, Investigation, Validation, Writing – review & editing. AH: Conceptualization, Writing – review & editing. AL: Data curation, Investigation, Validation, Writing – review & editing. RK: Data curation, Investigation, Validation, Writing – review & editing. CH: Data curation, Investigation, Validation, Writing – review & editing. LG: Data curation, Investigation, Validation, Writing – review & editing. GG: Data curation, Investigation, Validation, Writing – review & editing. SF: Data curation, Investigation, Validation, Writing – review & editing. SL: Data curation, Investigation, Validation, Writing – review & editing. MC-F: Data curation, Investigation, Validation, Writing – review & editing. VB: Data curation, Investigation, Validation, Writing – review & editing. KT: Conceptualization, Data curation, Funding acquisition, Investigation, Methodology, Project administration, Supervision, Validation, Writing – review & editing.
