# Supplementary material for: Changes in primary care visits for respiratory illness during the COVID-19 pandemic: a multinational study by the International Consortium of Primary Care Big Data Researchers (INTRePID)
Source: Front Med (Lausanne). 2024 Jun 17;11:1343646. doi: 10.3389/fmed.2024.1343646 (PMC11215147; doi:10.3389/fmed.2024.1343646)

Supplementary Material

Changes in primary care visits for respiratory illness during the COVID-19 pandemic: a multinational study by the International Consortium of Primary Care Big Data Researchers (INTRePID)

John M. Westfall, Angela Ortigoza Bonilla, María C. Lapadula, Paula L. Zingoni, William C. W. Wong, Knut A. Wensaas, Wilson D. Pace, Javier Silva-Valencia, Luciano F. Scattini, Amy P. P. Ng, Jo-Anne Manski-Nankervis, Zheng J. Ling, Zhuo Li, Adrian H. Heald, Adrian Laughlin, Robert S. Kristiansson, Christine M. Hallinan, Lay H. Goh, Gabriela Gaona, Signe Flottorp, Simon de Lusignan, María S. Cuba-Fuentes, Valborg Baste[16] and Karen Tu* on behalf of INTRePID.

*** Correspondence:**

Karen Tu. E-mail: k.tu@utoronto.ca

Table of Contents

Supplementary tables **3**

Table S1. INTRePID data sources and coverage by country 3

Table S2. Billing code systems used in INTRePID countries 5

Table S3. Upper Respiratory Tract Infections (URTI) diagnosis codes 6

Table S4. Lower Respiratory Tract Infections (LRTI) diagnosis codes 10

Table S5. Influenza-like illness diagnosis codes 13

Table S6. COVID-19 diagnosis codes 15

Table S7. Asthma diagnosis codes 16

Table S8. Chronic Obstructive Pulmonary Disease (COPD) diagnosis codes 18

Table S9. Respiratory allergies diagnosis codes 19

Table S10. Other respiratory disease diagnosis codes 20

Table S11. Assessment of COVID-19 care accessibility survey 22

Supplementary Figures **23**

Figure S1. Specific respiratory condition visits as a percentage of all respiratory visits in (A) Argentina, (B) Australia, (C) Canada, (D) China, (E) Norway, (F) Peru, (G) Singapore, (H) Sweden, and (I) United States. 23

Figure S2. Relative difference in mean monthly respiratory visits by category between the pre-pandemic and the pandemic periods in (A) Argentina, (B) Australia, (C) Canada, (D) China, (E) Norway, (F) Peru, (G) Singapore, (H) Sweden, and (I) United States. 24

.... **Error! Bookmark not defined.**

Figure S4. Upper Respiratory Tract Infections (URTI) visit rates as a percentage of total visits in (A) Argentina, (B) Australia, (C) Canada, (D) China, (E) Norway, (F) Peru, (G) Singapore, (H) Sweden, and (I) United States. 27

Figure S5. Lower Respiratory Tract Infections (LRTI) visit rates as a percentage of total visits in (A) Argentina, (B) Australia, (C) Canada, (D) China, (E) Norway, (F) Peru, (G) Singapore, (H) Sweden, and (I) United States. 28

Figure S6. COVID-19 visit rates as a percentage of total visits in (A) Argentina, (B) Australia, (C) Canada, (D) Norway, (E) Peru, (F) Singapore, (G) Sweden, and (H) United States. 29

Figure S7. Respiratory allergies visit rates as a percentage of total visits in (A) Argentina, (B) Australia, (C) Canada, (D) China, (E) Norway, (F) Peru, (G) Singapore, (H) Sweden, and (I) United States. 30

Figure S8. Chronic Obstructive Pulmonary Disease (COPD) visit rates as a percentage of total visits in (A) Argentina, (B) Australia, (C) Canada, (D) China, (E) Norway, (F) Peru, (G) Singapore, (H) Sweden, and (I) United States. 31

Figure S9. Asthma visit rates as a percentage of total visits in (A) Argentina, (B) Australia, (C) Canada, (D) China, (E) Norway, (F) Peru, (G) Singapore, (H) Sweden, and (I) United States. 32

Figure S10. Other respiratory diseases visit rates as a percentage of total visits in (A) Argentina, (B) Australia, (C) Canada, (D) China, (E) Norway, (F) Peru, (G) Singapore, (H) Sweden, and (I) United States. 33

Supplementary tables

Table S1. INTRePID data sources and coverage by country

Country	Period	Data Coverage	Data Source	Population
**Argentina**	2018-2021	All public level I healthcare centers in the Autonomous City of Buenos Aires: 46 Health and Community Care Centers (CESACs) and 39 Neighborhood Medical Centers (CMBs). ~ 600,000 patients.	Government of the Autonomous City of Buenos Aires	45.892.285. National Institute of Statistics and Censuses (INDEC).(1)
**Australia**	2018-2021	Convenience sample from the State of Victoria (1256 primary care physicians in 103 general practices). ~ 1.1 million patients.	Australian Patron Repository	26,821,557. Australian Bureau of Statistics (ABS).(2)
**Canada**	2018-2021	Convenience sample from the Province of Ontario. (392 primary care physicians in 95 clinics)..~400,000 patients.	University of Toronto Practice-Based Research Network (UTOPIAN) EMR database	36,991,981. Statistics Canada.(3)
**China**	2018-2021	Convenience sample from Hong Kong (13 primary care physicians and 3 psychotherapists from the University of Hong Kong Shen Zhen Hospital Family Medicine Clinic). ~40,000 patients.	University of Hong Kong Shen Zhen Hospital (HKUSZH) General Practice Department (GPD)	1,411,778,724. National Burau of Statistics of China.(4)
**Norway**	2018-2021	Full national coverage (all primary care centers). ~ 5 million patients.	Project 'COVID-19 outbreak in Norway – Epidemiology, health care utilization and primary care management – CONOPRI"	5,550,203. Statistics Norway (SSB).(5)
**Peru**	2019-2021	Full national coverage (8,263 public primary care centers from the Peruvian Comprehensive Health Insurance SIS). 23 million patients.	Peruvian Ministry of Health through the National Repository of Health Information (REUNIS acronym in Spanish)	31,237,385. National Institute of Statistics and Informatics (INEI).(6)
**Singapore**	2018-2021	Convenience sample from Singapore (886 primary care physicians from 6 public polyclinics). ~300,000 patients	National University Polyclinics (NUS) - Data Mart	5,917,600. Singapore Department of Statistics (DOS).(7)
**Sweden**	2018-2021	Full coverage of Upsala Region (150 primary care physicians). ~385,000 patients	Government of the Region of Uppsala	10,549,287. Statistics Sweden.(8)
**United States**	2018-2021	Convenience sample from the States of California, Texas, and Colorado (236 primary care physicians from DartNet primary care clinics). ~3 million patients	DartNet Institute	331,449,281. U.S. Census Bureau.(9)

Adapted from "Data sources from INTRePID countries (2018-2021)." Retrieved from https://www.intrepidprimarycare.org/resources

References

1. National Institute of Statistics and Censuses (INDEC). (2022). Census. Available from: https://www.indec.gob.ar/indec/web/Nivel3-Tema-2-41 (Accessed May 6, 2024)

2. Australian Bureau of Statistics (ABS). (2023). National, state and territory population. Available from: https://www.abs.gov.au/statistics/people/population/national-state-and-territory-population/sep-2023 (Accessed May 6, 2024)

3. Statistics Canada. (2022). Census Profile, 2021 Census of Population. Available from: https://www12.statcan.gc.ca/census-recensement/2021/dp-pd/prof/details/page.cfm?Lang=E&DGUIDList=2021A000011124&GENDERList=1,2,3&STATISTICList=1,4&HEADERList=0&SearchText=Canada (Accessed May 6, 2024)

4. National Burau of Statistics of China. (2021).Communiqué of the Seventh National Population Census (No. 3). Available from: https://www.stats.gov.cn/english/PressRelease/202105/t20210510_1817188.html (Accessed May 6, 2024)

5. Statistics Norway (SSB). (2023). Population. Available from: https://www.ssb.no/en/befolkning/folketall/statistikk/befolkning (Accessed May 6, 2024)

6. National Institute of Statistics and Informatics (INEI). (2023). Consult results of 2017 National Census. Available from: https://www.gob.pe/535-consultar-resultados-del-censo-nacional-2017 (Accessed May 6, 2024)

7. Singapore Department of Statistics (DOS). (2024). Population and Population Structure. Available from: http://www.singstat.gov.sg/find-data/search-by-theme/population/population-and-population-structure/latest-data (Accessed May 6, 2024)

8. Statistics Sweden. (2024). Available from: https://www.scb.se/en/ (Accessed May 6, 2024)

9. U.S. Census Bureau. (2024). U.S. and World Population Clock. Available from: https://www.census.gov/popclock/ (Accessed May 6, 2024)

Table S2. Billing code systems used in INTRePID countries

Country	Billing coding system
**Argentina**	ICD-10
**Australia**	SNOMED-CT
**Canada**	OHIP
**China**	ICD-10 / ICPC-2
**Norway**	ICPC-2
**Peru**	ICD-10/ ICD-10 CM
**Singapore**	ICD-10 AM
**Sweden**	ICD-10
**United States**	ICD-10/ ICD10-CM

Table S3. Upper Respiratory Tract Infections (URTI) diagnosis codes

System: ICD-10 / ICD-10 CM / ICD 10-AM

Code	Description	Found
A37.00	Whooping cough due to Bordetella pertussis without pneumonia	X
A37.80	Whooping cough due to other Bordetella species without pneumonia	X
A37.90	Whooping cough, unspecified species without pneumonia	X
B08.5	Enteroviral vesicular pharyngitis (herpangina)	X
H65	Nonsuppurative otitis media	
H65.1	Other acute nonsuppurative otitis media	X
H65.19	Other acute nonsuppurative otitis media	X
H65.9	Unspecified nonsuppurative otitis media	X
H65.90	Unspecified nonsuppurative otitis media, unspecified ear	X
H65.91	Unspecified nonsuppurative otitis media, right ear	X
H65.92	Unspecified nonsuppurative otitis media, left ear	X
H65.93	Unspecified nonsuppurative otitis media, bilateral	X
H66	Suppurative and unspecified otitis media	X
H66.0	Acute suppurative otitis media	X
H66.00	Acute suppurative otitis media without spontaneous rupture of ear drum	
H66.001	Acute suppurative otitis media without spontaneous rupture of ear drum, right ear	X
H66.002	Acute suppurative otitis media without spontaneous rupture of ear drum, left ear	X
H66.003	Acute suppurative otitis media without spontaneous rupture of ear drum, bilateral	X
H66.004	Acute suppurative otitis media without spontaneous rupture of ear drum, recurrent, right ear	X
H66.005	Acute suppurative otitis media without spontaneous rupture of ear drum, recurrent, left ear	X
H66.006	Acute suppurative otitis media without spontaneous rupture of ear drum, recurrent, bilateral	X
H66.007	Acute suppurative otitis media without spontaneous rupture of ear drum, recurrent, unspecified ear	
H66.009	Acute suppurative otitis media without spontaneous rupture of ear drum, unspecified ear	X
H66.01	Acute suppurative otitis media with spontaneous rupture of ear drum	
H66.011	Acute suppurative otitis media with spontaneous rupture of ear drum, right ear	X
H66.012	Acute suppurative otitis media with spontaneous rupture of ear drum, left ear	X
H66.013	Acute suppurative otitis media with spontaneous rupture of ear drum, bilateral	X
H66.014	Acute suppurative otitis media with spontaneous rupture of ear drum, recurrent, right ear	X
H66.015	Acute suppurative otitis media with spontaneous rupture of ear drum, recurrent, left ear	X
H66.016	Acute suppurative otitis media with spontaneous rupture of ear drum, recurrent, bilateral	
H66.017	Acute suppurative otitis media with spontaneous rupture of ear drum, recurrent, unspecified ear	
H66.019	Acute suppurative otitis media with spontaneous rupture of ear drum, unspecified ear	
H66.3	Other chronic suppurative otitis media	X
H66.4	Suppurative otitis media, unspecified	X
H66.40	Suppurative otitis media, unspecified, unspecified ear	
H66.41	Suppurative otitis media, unspecified, right ear	X

Table S3. Upper Respiratory Tract Infections (URTI) diagnosis codes (continued)

System: ICD-10 / ICD-10 CM / ICD 10-AM

Code	Description	Found
H66.42	Suppurative otitis media, unspecified, left ear	X
H66.43	Suppurative otitis media, unspecified, bilateral	X
H66.9	Otitis media, unspecified	X
H66.90	Otitis media, unspecified, unspecified ear	X
H66.91	Otitis media, unspecified, right ear	X
H66.92	Otitis media, unspecified, left ear	X
H66.93	Otitis media, unspecified, bilateral	X
J00	Acute nasopharyngitis common cold	X
J01	Acute sinusitis	X
J01.0	Acute maxillary sinusitis	X
J01.00	Acute maxillary sinusitis, unspecified	X
J01.01	Acute recurrent maxillary sinusitis	X
J01.1	Acute frontal sinusitis	X
J01.10	Acute frontal sinusitis, unspecified	X
J01.11	Acute recurrent frontal sinusitis	X
J01.2	Acute ethmoidal sinusitis	X
J01.20	Acute ethmoidal sinusitis, unspecified	X
J01.21	Acute recurrent ethmoidal sinusitis	X
J01.3	Acute sphenoidal sinusitis	X
J01.30	Acute sphenoidal sinusitis, unspecified	X
J01.31	Acute recurrent sphenoidal sinusitis	X
J01.4	Acute pansinusitis	X
J01.40	Acute pansinusitis, unspecified	X
J01.41	Acute recurrent pansinusitis	X
J01.8	Other acute sinusitis	X
J01.80	Other acute sinusitis	X
J01.81	Other acute recurrent sinusitis	X
J01.9	Acute sinusitis, unspecified	X
J01.90	Acute sinusitis, unspecified	X
J01.91	Acute recurrent sinusitis, unspecified	X
J02	Acute pharyngitis	X
J02.0	Streptococcal pharyngitis	X
J02.8	Acute pharyngitis due to other specified organisms	X
J02.9	Acute pharyngitis, unspecified	X
J03	Acute tonsillitis	X
J03.0	Streptococcal tonsillitis	X
J03.00	Acute streptococcal tonsillitis, unspecified	X
J03.01	Acute recurrent streptococcal tonsillitis	X
J03.8	Acute tonsillitis due to other specified organisms	X
J03.80	Acute tonsillitis due to other specified organisms	X
J03.81	Acute recurrent tonsillitis due to other specified organisms	X
J03.9	Acute tonsillitis, unspecified	X
J03.90	Acute tonsillitis, unspecified	X
J03.91	Acute recurrent tonsillitis, unspecified	X
J04.0	Acute laryngitis	X
J04.1	Acute tracheitis	X
J04.10	Acute tracheitis without obstruction	X
J04.2	Acute laryngotracheitis	X
J05.0	Acute obstructive laryngitis [croup]	X

Table S3. Upper Respiratory Tract Infections (URTI) diagnosis codes (continued)

System: ICD-10 / ICD-10 CM / ICD 10-AM

Code	Description	Found
J05.1	Acute epiglottitis	X
J05.10	Acute epiglottitis without obstruction	X
J06	Acute upper respiratory infections of multiple and unspecified sites	X
J06.0	Acute laryngopharyngitis	X
J06.8	Other acute upper respiratory infections of multiple sites	X
J06.9	Acute upper respiratory infection, unspecified	X

System: SNOMED CT

Code	Description	Found
1532007	Viral pharyngitis	X
3110003	Acute otitis media	
5028002	Acute pansinusitis	
6965008	Subacute nonsuppurative otitis media	
11134001	Acute suppuration of sphenoidal sinus	
14852000	Myringitis	
15805002	Acute sinusitis	
16664009	Malignant otitis media	
17357005	Acute suppuration of frontal sinus	
19021002	Haemophilus influenzae otitis media	
23884004	Acute suppuration of maxillary sinus	
29350000	Catarrhal otitis media	
32760002	Exudative otitis media	
36971009	Sinusitis	X
39288006	Purulent otitis media	
45913009	Laryngitis	X
54150009	Upper respiratory tract infection	X
54398005	Acute upper respiratory infection	
63140003	Acute suppuration of ethmoidal sinus	
65363002	Otitis media	X
67832005	Acute ethmoidal sinusitis	
68272006	Acute maxillary sinusitis	
71186008	Croup	X
75498004	Acute bacterial sinusitis	
77919000	Acute sphenoidal sinusitis	
80600003	Acute suppuration of nasal sinus	
82272006	Common cold	X
82297005	Congestion of nasal sinus	X
85940005	Tuberculous otitis media	
89765009	Rhinosinusitis	X
90176007	Tonsillitis	X
91038008	Acute frontal sinusitis	
155516001	Acute upper respiratory infection	
186659004	Herpangina	
194237006	Nonsuppurative otitis media with eustachian tube disorder	
194286008	Bilateral suppurative otitis media	

Table S3. Upper Respiratory Tract Infections (URTI) diagnosis codes (continued)

System: SNOMED CT

Code	Description	Found
195704001	Other acute upper respiratory infections	
195705000	Acute upper respiratory infection	
195708003	Recurrent upper respiratory tract infection	
196195006	Acute upper respiratory infection	
232256002	Otitis media with effusion sanguineous	
232342002	Common cold	
232391008	Recurrent acute sinusitis	
275481002	Nonsuppurative otitis media	
281794004	Viral upper respiratory tract infection	
312118003	Bacterial upper respiratory infection	X
312218008	Infective otitis media	
359609001	Acute secretory otitis media	
405737000	Pharyngitis	X
431231008	Acute rhinosinusitis	
703469002	Bacterial otitis media	
715882005	Severe acute respiratory syndrome of upper respiratory tract	
721742004	Otitis media caused by Streptococcus pneumoniae	
725916003	Acute sinusitis caused by fungus	
725917007	Acute sinusitis caused by virus	
789057009	Tuberculosis of middle ear	
7361000119105	Perforation of tympanic membrane due to otitis media	
1083431000119100	Bilateral chronic suppurative otitis media of middle ears	
1089341000119100	Otitis media of left ear	
1089661000119108	Suppurative otitis media of left ear	
1091951000119104	Otitis media of right ear	
**1** 1092271000119106	Suppurative otitis media of right ear	

System: ICPC-2

Code	Description	Found
H71	Acute otitis media/myringitis	X
R07	Sneezing/nasal congestion	X
R71	Whooping cough	X
R72	Strep throat	X
R74	Upper respiratory infection acute	X
R75	Sinusitis acute/chronic	X
R76	Tonsillitis acute	X
R77	Laryngitis/tracheitis acute	X

System: OHIP

Code	Description	Found
382	Otitis Media Suppurative	X
460	Acute nasopharyngitis, common cold, Upper Respiratory	X
461	Acute sinusitis	X
464	Acute laryngitis, tracheitis, croup, epiglottis	X

Table S4. Lower Respiratory Tract Infections (LRTI) diagnosis codes

System: ICD-10 / ICD-10 CM / ICD 10-AM

Code	Description	Found
J12	Viral pneumonia, not elsewhere classified	X
J12.0	Adenoviral pneumonia	X
J12.1	Respiratory syncytial virus pneumonia	X
J12.2	Parainfluenza virus pneumonia	X
J12.3	Human metapneumovirus pneumonia	X
J12.8	Other viral pneumonia	X
J12.89	Other viral pneumonia	X
J12.9	Viral pneumonia, unspecified	X
J13	Pneumonia due to Streptococcus pneumoniae	X
J14	Pneumonia due to Hemophilus influenzae	X
J15	Bacterial pneumonia, not elsewhere classified	X
J15.0	Pneumonia due to Klebsiella pneumoniae	X
J15.1	Pneumonia due to Pseudomonas	X
J15.2	Pneumonia due to staphylococcus	X
J15.20	Pneumonia due to staphylococcus, unspecified	X
J15.21	Pneumonia due to staphylococcus aureus	
J15.211	Pneumonia due to Methicillin susceptible Staphylococcus aureus	
J15.212	Pneumonia due to Methicillin resistant Staphylococcus aureus	
J15.29	Pneumonia due to other staphylococcus	
J15.3	Pneumonia due to streptococcus, group B	X
J15.4	Pneumonia due to other streptococci	X
J15.5	Pneumonia due to Escherichia coli	X
J15.6	Pneumonia due to other Gramnegative bacteria	X
J15.7	Pneumonia due to Mycoplasma pneumoniae	X
J15.8	Pneumonia due to other specified bacteria	X
J15.9	Unspecified bacterial pneumonia	X
J16	Pneumonia due to other infectious organisms, not elsewhere classified	
J16.0	Chlamydial pneumonia	X
J16.8	Pneumonia due to other specified infectious organisms	X
J17	Pneumonia in diseases classified elsewhere	X
J17.0	Pneumonia in bacterial diseases classified elsewhere	X
J17.1	Pneumonia in viral diseases classified elsewhere	X
J17.2	Pneumonia in mycoses	X
J17.3	Pneumonia in parasitic diseases	X
J17.8	Pneumonia in other diseases classified elsewhere	X
J18	Pneumonia, unspecified organism	X
J18.0	Bronchopneumonia, unspecified organism	X
J18.1	Lobar pneumonia, unspecified organism	X
J18.2	Hypostatic pneumonia, unspecified organism	X
J18.8	Other pneumonia, unspecified organism	X
J18.9	Pneumonia, unspecified organism	X
J20	Acute bronchitis	X
J20.0	Acute bronchitis due to Mycoplasma pneumoniae	X
J20.1	Acute bronchitis due to Hemophilus influenzae	X
J20.2	Acute bronchitis due to streptococcus	X
J20.3	Acute bronchitis due to coxsackievirus	X
J20.4	Acute bronchitis due to parainfluenza virus	X
J20.5	Acute bronchitis due to respiratory syncytial virus	X

Table S4. Lower Respiratory Tract Infections (LRTI) diagnosis codes (continued)

System: ICD-10 / ICD-10 CM / ICD 10-AM

Code	Description	Found
J20.6	Acute bronchitis due to rhinovirus	X
J20.7	Acute bronchitis due to echovirus	X
J20.8	Acute bronchitis due to other specified organisms	X
J20.9	Acute bronchitis, unspecified	X
J21	Acute bronchiolitis	X
J21.0	Acute bronchiolitis due to respiratory syncytial virus	X
J21.1	Acute bronchiolitis due to metapneumovirus	X
J21.8	Acute bronchiolitis due to other specified organisms	X
J21.9	Acute bronchiolitis, unspecified	X
J22	Unspecified acute lower respiratory infection	X
J47.0	Bronchiectasis with acute lower respiratory infection	X
J47.1	Bronchiectasis with (acute) exacerbation	X
J20.6	Acute bronchitis due to rhinovirus	X
J20.7	Acute bronchitis due to echovirus	X
J20.8	Acute bronchitis due to other specified organisms	X
J20.9	Acute bronchitis, unspecified	X
J21	Acute bronchiolitis	X
J21.0	Acute bronchiolitis due to respiratory syncytial virus	X
J21.1	Acute bronchiolitis due to metapneumovirus	X
J21.8	Acute bronchiolitis due to other specified organisms	X
J21.9	Acute bronchiolitis, unspecified	X
J22	Unspecified acute lower respiratory infection	X
J47.0	Bronchiectasis with acute lower respiratory infection	X
J47.1	Bronchiectasis with (acute) exacerbation	X

System: SNOMED CT

Code	Description	Found
4120002	Bronchiolitis	X
5875001	Acute bronchitis with obstruction	
10509002	Acute bronchitis	X
16146001	Viral bronchitis	X
27475006	Parainfluenza virus bronchitis	
29591002	Purulent bronchitis	
32398004	Bronchitis	X
35301006	Acute tracheobronchitis	
50417007	Acute lower respiratory tract infection	X
50804000	Catarrhal pneumonia	
55679008	Peri-bronchial pneumonia	
57702005	Unresolved pneumonia	
68409003	Organized pneumonia	
80257001	Acute bronchitis with bronchospasm	
85469005	Hypostatic pneumonia	
111849006	Adenoviral bronchitis	

Table S4. Lower Respiratory Tract Infections (LRTI) diagnosis codes (continued)

System: SNOMED CT

Code	Description	Found
123591006	Confluent pneumonia	
195717003	Acute purulent bronchitis	
196112005	Abscess of lung with pneumonia	
233598009	Acute bacterial bronchitis	
233601004	Acute viral bronchitis	
233604007	Pneumonia	X
233617005	Hemorrhagic pneumonia	
275498002	Respiratory tract infection	X
278516003	Lobar pneumonia	
312342009	Infective pneumonia	
312371005	Acute infective bronchitis	
312400008	Acute infective tracheobronchitis	
371072008	Post-obstructive pneumonia	
385093006	Community acquired pneumonia	
396285007	Bronchopneumonia	
407671000	Bilateral pneumonia	
714203003	Acute bronchitis co-occurrent with bronchiectasis	
785728005	Acute bronchitis co-occurrent with wheeze	X
1163489008	Human metapneumovirus bronchitis	
106001000119101	Chronic obstructive lung disease co-occurrent with acute bronchitis	
10625751000119106	Bronchopneumonia due to virus	

System: ICPC-2

Code	Description	Found
R78	Acute bronchitis/bronchiolitis	X
R81	Pneumonia	X
R83	Respiratory infection other	X

System: OHIP

Code	Description	Found
466	Acute bronchitis	X
486	Pneumonia all types	X

Table S5. Influenza-like illness diagnosis codes

System: ICD-10 / ICD-10 CM / ICD 10-AM

Code	Description	Found
J09	Influenza due to certain identified influenza viruses	X
J09.X	Influenza due to identified novel influenza A virus	X
J09.X1	Influenza due to identified novel influenza A virus with pneumonia	X
J09.X2	Influenza due to identified novel influenza A virus with other respiratory manifestations	X
J09.X3	Influenza due to identified novel influenza A virus with gastrointestinal manifestations	X
J09.X9	Influenza due to identified novel influenza A virus with other manifestations	X
J10	Influenza due to other identified influenza virus	X
J10.0	Influenza due to other identified influenza virus with pneumonia	X
J10.00	Influenza due to other identified influenza virus vith unspecified type of pneumonia	X
J10.01	Influenza due to other identified influenza virus with the same other identified influenza virus pneumonia	X
J10.08	Influenza due to other identified influenza virus vith other specified pneumonia	X
J10.1	Influenza due to other identified influenza virus with other respiratory manifestations	X
J10.2	Influenza due to other identified influenza virus with gastrointestinal manifestations	X
J10.8	Influenza due to other identified influenza virus with other manifestations	X
J10.81	Influenza due to other identified influenza virus with encephalopathy	X
J10.82	Influenza due to other identified influenza virus with myocarditis	
J10.83	Influenza due to other identified influenza virus with otitis media	
J10.89	Influenza due to other identified influenza virus with other manifestations	X
J11	Influenza due to unidentified influenza virus	X
J11.0	Influenza due to unidentified influenza virus with pneumonia	X
J11.00	Influenza due to unidentified influenza virus with unspecified type of pneumonia	X
J11.08	Influenza due to unidentified influenza virus with specified pneumonia	X
J11.1	Influenza due to unidentified influenza virus with other respiratory manifestations	X
J11.2	Influenza due to unidentified influenza virus with gastrointestinal manifestations	X
J11.8	Influenza due to unidentified influenza virus with other manifestations	X
J11.81	Influenza due to unidentified influenza virus with encephalopathy	
J11.82	Influenza due to unidentified influenza virus with myocarditis	
J11.83	Influenza due to unidentified influenza virus with otitis media	X
J11.89	Influenza due to unidentified influenza virus with other manifestations	X

System: SNOMED CT

Code	Description	Found
24662006	Influenza due to Influenza B virus	
41269000	Influenzal bronchopneumonia	
43692000	Influenzal acute upper respiratory infection	
61700007	Influenza with nonrespiratory manifestation	
74644004	Influenza with encephalopathy	
81524006	Influenza due to Influenza C virus	
95891005	Influenza like illness	X
195878008	Pneumonia and influenza	
195923003	Influenza with laryngitis	
195924009	Influenza with pharyngitis	
408687004	Healthcare associated influenza disease	
442438000	Influenza due to Influenza A virus	

Table S5. Influenza-like illness diagnosis codes (continued)

System: SNOMED CT

Code	Description	Found
719590007	Influenza caused by seasonal influenza virus	
719865001	Influenza caused by pandemic influenza virus	
772839003	Pneumonia caused by Influenza A virus	
16311000119108	Pneumonia due to influenza	
328531000119104	Upper respiratory tract infection due to Influenza A	
434931000124106	Pneumonia due to Influenza A virus subtype H1N1	
10624951000119108	Otitis media due to influenza	
10629191000119100	Bronchiolitis caused by influenza virus	
10685111000119102	Upper respiratory tract infection due to Influenza	

System: ICPC-2

Code	Description	Found
R80	Influenza	X

System: OHIP

Code	Description	Found
487	Influenza	X

Table S6. COVID-19 diagnosis codes

System: ICD-10 / ICD-10 CM / ICD 10-AM

Code	Description	Found
B34.2	Coronavirus infection, unspecified	X
B97	Coronavirus as the cause of diseases classified elsewhere	
B97.2	Coronavirus as the cause of diseases classified elsewhere	X
B97.21	SARS associated coronavirus causing diseases classified elsewhere	X
B97.29	Other coronavirus as the cause of diseases classified elsewhere	X
J12.81	Pneumonia due to SARS-associated coronavirus	X
J12.82	Pneumonia due to coronavirus disease 2019	X
U07	COVID-19	
U07.1	COVID-19, identified	X
U07.2	COVID-19, not identified	

System: SNOMED TC

Code	Description	Found
840539006	COVID-19	X
840544004	Suspected COVID-19	
870588003	Sepsis due to disease caused by COVID-19	
1119302008	Acute COVID-19	
1240521000000100	Otitis media due to COVID-19	
1240541000000107	Infection of upper respiratory tract caused by SARS-CoV2	
67481402100011900[illegible]	Acute respiratory distress syndrome due to COVID-19	

System: ICPC-2

Code	Description	Found
R991	COVID-19 suspected	X
R992	COVID-19 confirmed	X

System: OHIP

Code	Description	Found
080	Coronavirus (suspected and confirmed)	X

Table S7. Asthma diagnosis codes

System: ICD-10 / ICD-10 CM / ICD 10-AM

Code	Description	Found
J45	Asthma	X
J45.0	Asthma predominantly allergic	X
J45.1	Non allergic asthma	X
J45.2	Mild intermittent asthma	X
J45.20	Mild intermittent asthma, uncomplicated	X
J45.21	Mild intermittent asthma with (acute) exacerbation	X
J45.22	Mild intermittent asthma with status asthmaticus	X
J45.3	Mild persistent asthma	X
J45.30	Mild persistent asthma, uncomplicated	X
J45.31	Mild persistent asthma with (acute) exacerbation	X
J45.32	Mild persistent asthma with status asthmaticus	X
J45.4	Moderate persistent asthma	X
J45.40	Moderate persistent asthma, uncomplicated	X
J45.41	Moderate persistent asthma with (acute) exacerbation	X
J45.42	Moderate persistent asthma with status asthmaticus	X
J45.5	Severe persistent asthma	X
J45.50	Severe persistent asthma, uncomplicated	X
J45.51	Severe persistent asthma with (acute) exacerbation	X
J45.52	Severe persistent asthma with status asthmaticus	X
J45.8	Mixed asthma	X
J45.9	Asthma, unspecified	X
J45.90	Unspecified asthma	X
J45.901	Unspecified asthma with (acute) exacerbation	X
J45.902	Unspecified asthma with status asthmaticus	X
J45.909	Unspecified asthma, uncomplicated	X
J45.91	Chronic obstructive syndrome. Wheezing, bronchial hyperactivity	X
J45.99	Other asthma	
J45.990	Exercise induced bronchospasm	X
J45.991	Cough variant asthma	X
J45.998	Other asthma	X

System: SNOMED CT

Code	Description	Found
31387002	Exercise induced asthma	
55570000	Asthma without status asthmaticus	
195967001	Asthma	X
195977004	Mixed asthma	
370218001	Mild asthma	
370219009	Moderate asthma	
370221004	Severe asthma	
389145006	Allergic asthma	X
405944004	Asthmatic bronchitis	X
409663006	Cough variant asthma	
425969006	Exacerbation of intermittent asthma	
426656000	Severe persistent asthma	
427603009	Intermittent asthma	
427679007	Mild intermittent asthma	
734905008	Moderate acute exacerbation of asthma	

Table S7. Asthma diagnosis codes (continued)

System: SNOMED CT

Code	Description	Found
762521001	Exacerbation of allergic asthma	X
782520007	Exacerbation of allergic asthma due to infection	X
1741000119102	Intermittent asthma, uncontrolled	
675871000119106	Mild persistent allergic asthma	
10674991000119104	Intermittent allergic asthma	
10675391000119100	Severe controlled persistent asthma	
10675751000119107	Severe uncontrolled persistent asthma	

System: ICPC-2

Code	Description	Found
R96	Asthma	X

System: OHIP

Code	Description	Found
493	Asthma, allergic bronchitis	X

Table S8. Chronic Obstructive Pulmonary Disease (COPD) diagnosis codes

System: ICD-10 / ICD-10 CM / ICD 10-AM

Code	Description	Found
J40	Bronchitis not specified as acute or chronic	X
J41	Simple and mucopurulent chronic bronchitis	
J41.0	Simple chronic bronchitis	X
J41.1	Mucopurulent chronic bronchitis	X
J41.8	Mixed simple and mucopurulent chronic bronchitis	X
J42	Unspecified chronic bronchitis	X
J43	Emphysema	X
J43.0	Unilateral pulmonary emphysema MacLeod's syndrome	X
J43.1	Panlobular emphysema	X
J43.2	Centrilobular emphysema	X
J43.8	Other emphysema	X
J43.9	Emphysema, unspecified	X
J44	Other chronic obstructive pulmonary disease	X
J44.0	Chronic obstructive pulmonary disease with (acute) lower respiratory infection	X
J44.1	Chronic obstructive pulmonary disease with (acute) exacerbation	X
J44.8	Other specified chronic obstructive pulmonary disease	X
J44.9	Chronic obstructive pulmonary disease, unspecified	X

System: SNOMED CT

Code	Description	Found
13645005	Chronic obstructive pulmonary disease	X
49158009	Emphysema	
63480004	Chronic bronchitis	
125294002	Acute emphysema	
125295001	Chronic emphysema	
196001008	Chronic obstructive pulmonary disease with acute lower respiratory infection	
285381006	Acute infective exacerbation of copd	X
313296004	Mild chronic obstructive pulmonary disease	
313297008	Moderate chronic obstructive pulmonary disease	
313299006	Severe chronic obstructive pulmonary disease	

System: ICPC-2

Code	Description	Found
R79	Chronic bronchitis	X
R95	Chronic obstructive pulmonary disease	X

System: OHIP

Code	Description	Found
491	Chronic bronchitis	X
492	Emphysema	X
496	Other chronic obstructive pulmonary disease	X

Table S9. Respiratory allergies diagnosis codes

System: ICD-10 / ICD-10 CM / ICD 10-AM

Code	Description	Found
J30	Vasomotor and allergic rhinitis	
J30.0	Vasomotor rhinitis	X
J30.1	Allergic rhinitis due to pollen	X
J30.2	Other seasonal allergic rhinitis	X
J30.3	Other allergic rhinitis	X
J30.4	Allergic rhinitis, unspecified	X
J30.5	Allergic rhinitis due to food	X
J30.8	Other allergic rhinitis	X
J30.81	Allergic rhinitis due to animal (cat) (dog) hair and dander	X
J30.89	Other allergic rhinitis	X
J30.9	Allergic rhinitis, unspecified	X

System: SNOMED CT

Code	Description	Found
21719001	Allergic rhinitis due to pollen	
61582004	Allergic rhinitis	X
91925003	Allergic rhinitis due to animals	
232353008	Perennial allergic rhinitis with seasonal variation	
232430006	Recurrent allergic croup	
367498001	Seasonal allergic rhinitis	
405720007	Allergic bronchitis	
429195002	Allergic rhinitis due to animal dander	
441978001	Allergic rhinitis due to food	
446096008	Perennial allergic rhinitis	
449729000	Allergic rhinitis due to house dust mite	
10629391000119103	Allergic rhinitis caused by feathers	
10629431000119108	Allergic rhinitis caused by insects	

System: ICPC-2

Code	Description	Found
R97	Allergic rhinitis	X

System: OHIP

Code	Description	Found
477	Allergic rhinitis, hay fever	X

Table S10. Other respiratory disease diagnosis codes

System: ICD-10 / ICD-10 CM / ICD 10-AM

Code	Description	Found
A15	Respiratory tuberculosis	X
A15.0	Tuberculosis of lung	X
A15.1	Tuberculosis of the lung, confirmed by culture only	X
A15.2	Tuberculosis of lung	X
A15.3	Tuberculosis of lung	X
A15.4	Tuberculosis of intrathoracic lymph nodes	X
A15.5	Tuberculosis of larynx, trachea and bronchus	X
A15.6	Tuberculous pleurisy	X
A15.7	Primary respiratory tuberculosis	X
A15.8	Other respiratory tuberculosis	X
A15.9	Respiratory tuberculosis unspecified	X
A16	Respiratory tuberculosis not confirmed bacteriologically, molecularly or histologically	
A16.0	Tuberculosis of the lung, not confirmed bacteriologically, molecularly or histologically	X
A16.1	Tuberculosis of the lung, not confirmed bacteriologically, molecularly or histologically	X
A16.2	Tuberculosis of the lung, without mention of bacteriological, molecular of histological confirmation	X
A16.5	Tuberculous pleurisy, without mention of bacteriological or histological confirmation	X
A16.7	Primary respiratory tuberculosis , without mention of bacteriological, molecular or histological confirmation	X
A16.8	Other respiratory tuberculosis, without mention of bacteriological, molecular or histological confirmation	X
A16.9	Respiratory tuberculosis unspecified, without mention of bacteriological, molecular of histological confirmation	X
J98	Other respiratory disorders	
J98.8	Other specified respiratory disorders	X
J98.9	Respiratory disorder, unspecified	X
J99	Respiratory disorders in diseases classified elsewhere	X
R05.2	Subacute cough	X
R05.3	Chronic cough	X
R05.8	Other specified cough	X
R05.9	Cough, unspecified	X

System: SNOMED CT

Code	Description	Found
49727002	Cough	X
56717001	Tuberculosis	
445241004	Postviral cough	X
707224005	Severe acute respiratory infection	

Table S10. Other respiratory disease diagnosis codes (continued)

System: ICPC-2

Code	Description	Found
R05	Cough	X
R70	Tuberculosis	
R99	Respiratory disease other	X

System: OHIP

Code	Description	Found
011	Pulmonary tuberculosis	X
786	Respiratory system signs and symptoms not yet diagnosed, cough, dyspnea, dysuria, epistaxis, hemoptysis, hyperventilation, oligouria, shortness of breath,	X

Table S11. Assessment of COVID-19 care accessibility survey

Please specify the frequency of occurrence for each scenario related to the assessment of potential COVID-19 patients in your country for the years 2020 and 2021, selecting only one option per year.

	Question	2020	2021
**1**	How frequently were virtual assessments for potential COVID-19 patients conducted in your country during the years 2020 and 2021?	☐ Always<br>☐ Common<br>☐ Sometimes<br>☐ Occasionally<br>☐ Never	☐ Always<br>☐ Common<br>☐ Sometimes<br>☐ Occasionally<br>☐ Never
**2**	How often were primary care offices utilized for assessing potential COVID-19 patients in your country in the years 2020 and 2021?	☐ Always<br>☐ Common<br>☐ Sometimes<br>☐ Occasionally<br>☐ Never	☐ Always<br>☐ Common<br>☐ Sometimes<br>☐ Occasionally<br>☐ Never
**3**	What was the frequency of assessment center usage for potential COVID-19 patients in your country during the years 2020 and 2021?	☐ Always<br>☐ Common<br>☐ Sometimes<br>☐ Occasionally<br>☐ Never	☐ Always<br>☐ Common<br>☐ Sometimes<br>☐ Occasionally<br>☐ Never
**4**	How frequently were after-hours clinics utilized for assessing potential COVID-19 patients in your country in the years 2020 and 2021?	☐ Always<br>☐ Common<br>☐ Sometimes<br>☐ Occasionally<br>☐ Never	☐ Always<br>☐ Common<br>☐ Sometimes<br>☐ Occasionally<br>☐ Never
**5**	What was the frequency of utilization of emergency departments for assessing potential COVID-19 patients in your country during the years 2020 and 2021?	☐ Always<br>☐ Common<br>☐ Sometimes<br>☐ Occasionally<br>☐ Never	☐ Always<br>☐ Common<br>☐ Sometimes<br>☐ Occasionally<br>☐ Never
**6**	How often were home visits conducted for potential COVID-19 patients in your country during the years 2020 and 2021?	☐ Always<br>☐ Common<br>☐ Sometimes<br>☐ Occasionally<br>☐ Never	☐ Always<br>☐ Common<br>☐ Sometimes<br>☐ Occasionally<br>☐ Never

Supplementary Figures

Figure S1. Specific respiratory condition visits as a percentage of all respiratory visits in (A) Argentina, (B) Australia, (C) Canada, (D) China, (E) Norway, (F) Peru, (G) Singapore, (H) Sweden, and (I) United States. Stack column charts display monthly rates for different categories of respiratory visits. The rates were calculated by dividing the number of specific respiratory condition visits by the total number of respiratory visits to primary care services within each month and are expressed as percentages (%).

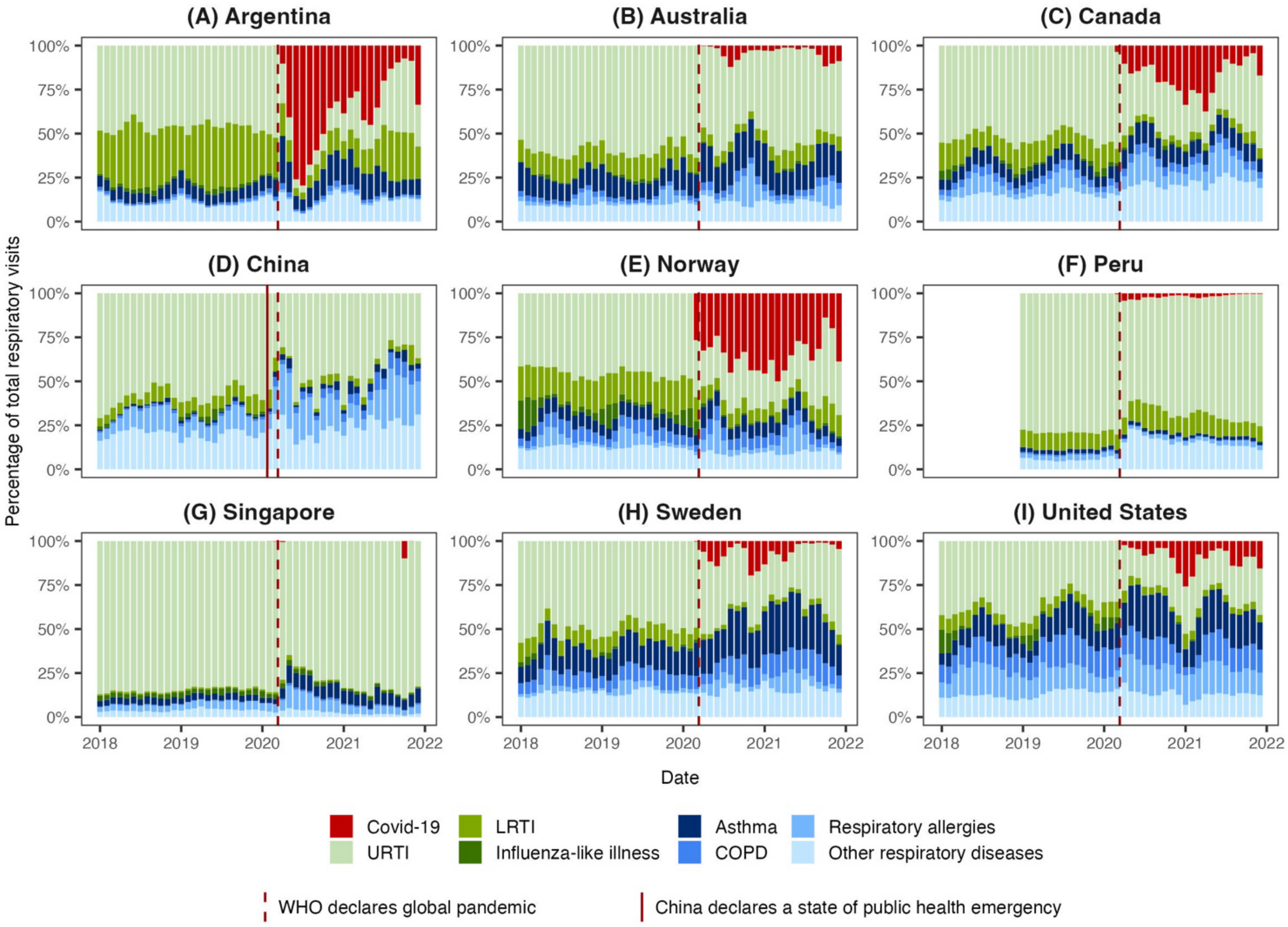

Figure S2. Relative difference in mean monthly respiratory visits by category between the pre-pandemic and the pandemic periods in (A) Argentina, (B) Australia, (C) Canada, (D) China, (E) Norway, (F) Peru, (G) Singapore, (H) Sweden, and (I) United States. Differences in mean monthly visits, 95% confidence intervals, and p-values were calculated using Welch's t-test. The dots represent the relative difference in means between the two periods and are expressed as percentages (%). The error bars denote the 95% confidence intervals. Significance levels are indicated as follows: *p < 0.05; **p < 0.01; ***p < 0.001.

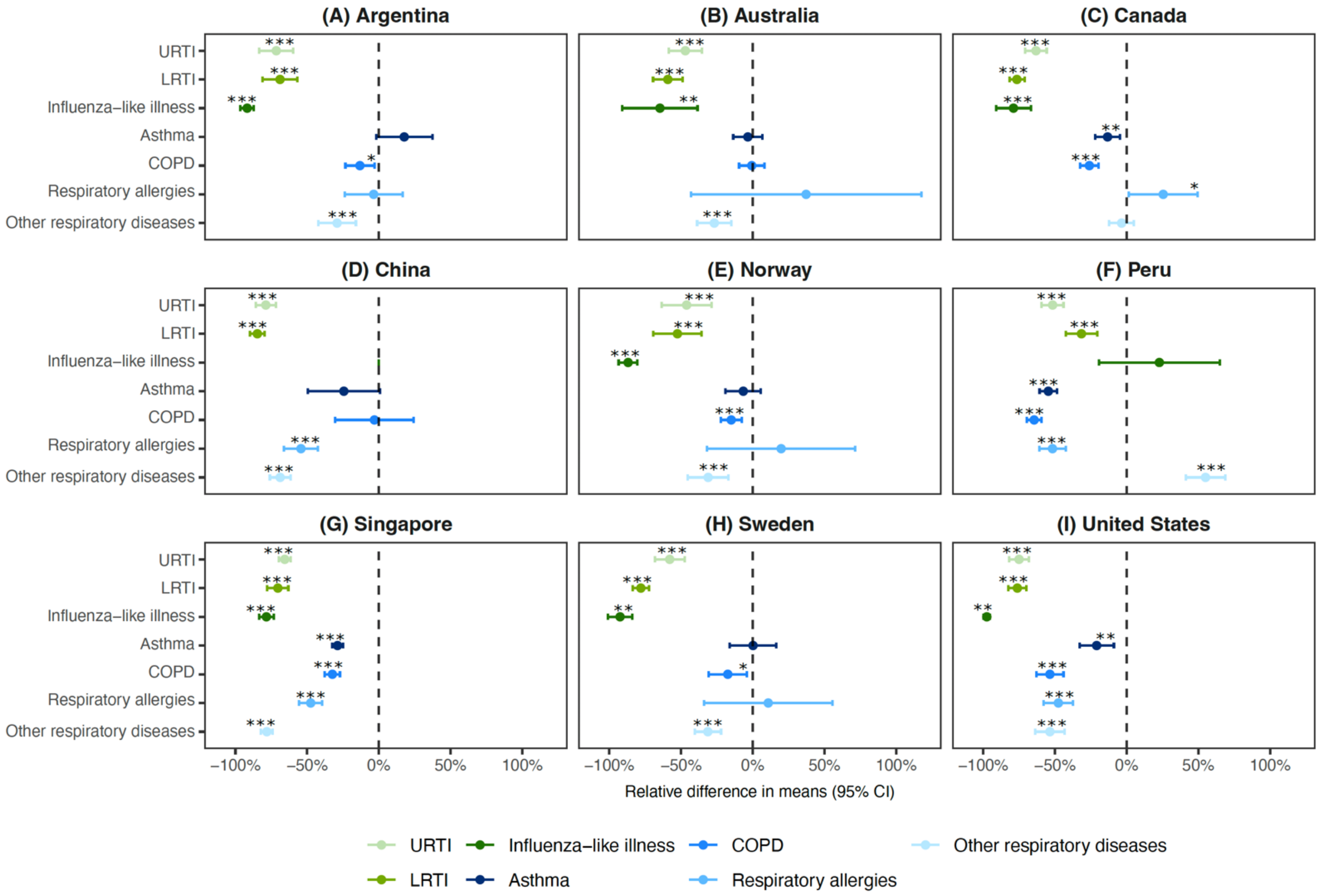

Figure S3. Influenza-like illness visit rates as a percentage of total visits in (A) Argentina, (B) Australia, (C) Canada, (D) China, (E) Norway, (F) Peru, (G) Singapore, (H) Sweden, and (I) United States. Trendlines show the monthly rates of influenza-like illness visits over time. The rates were calculated by dividing the number of influenza-like illness visits by the total visits to primary care services within each month and are expressed as percentages (%).

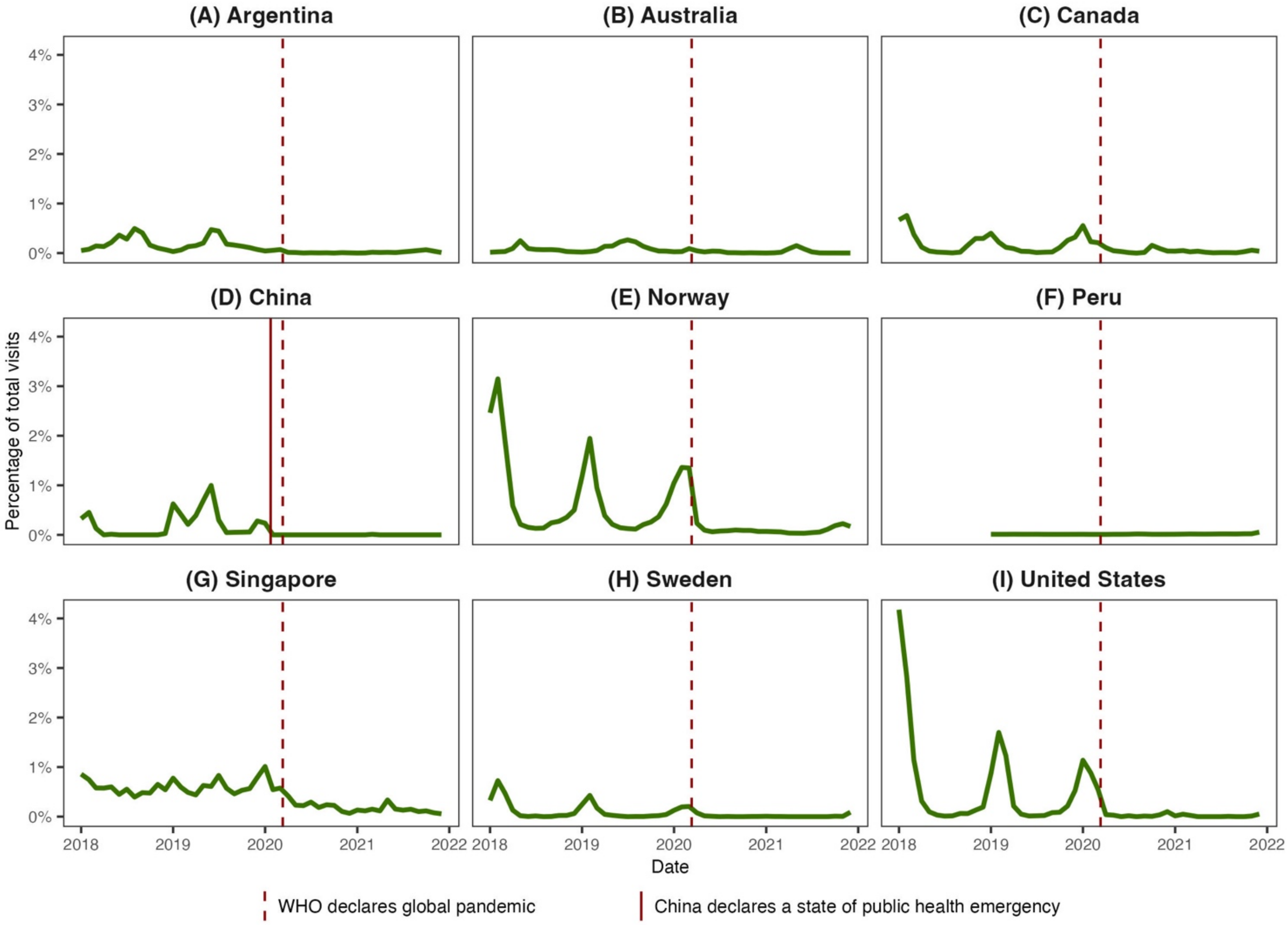

Figure S4. Upper Respiratory Tract Infections (URTI) visit rates as a percentage of total visits in (A) Argentina, (B) Australia, (C) Canada, (D) China, (E) Norway, (F) Peru, (G) Singapore, (H) Sweden, and (I) United States. Trendlines show the monthly rates of URTI visits over time. The rates were calculated by dividing the number of URTI visits by the total visits to primary care services within each month and are expressed as percentages (%).

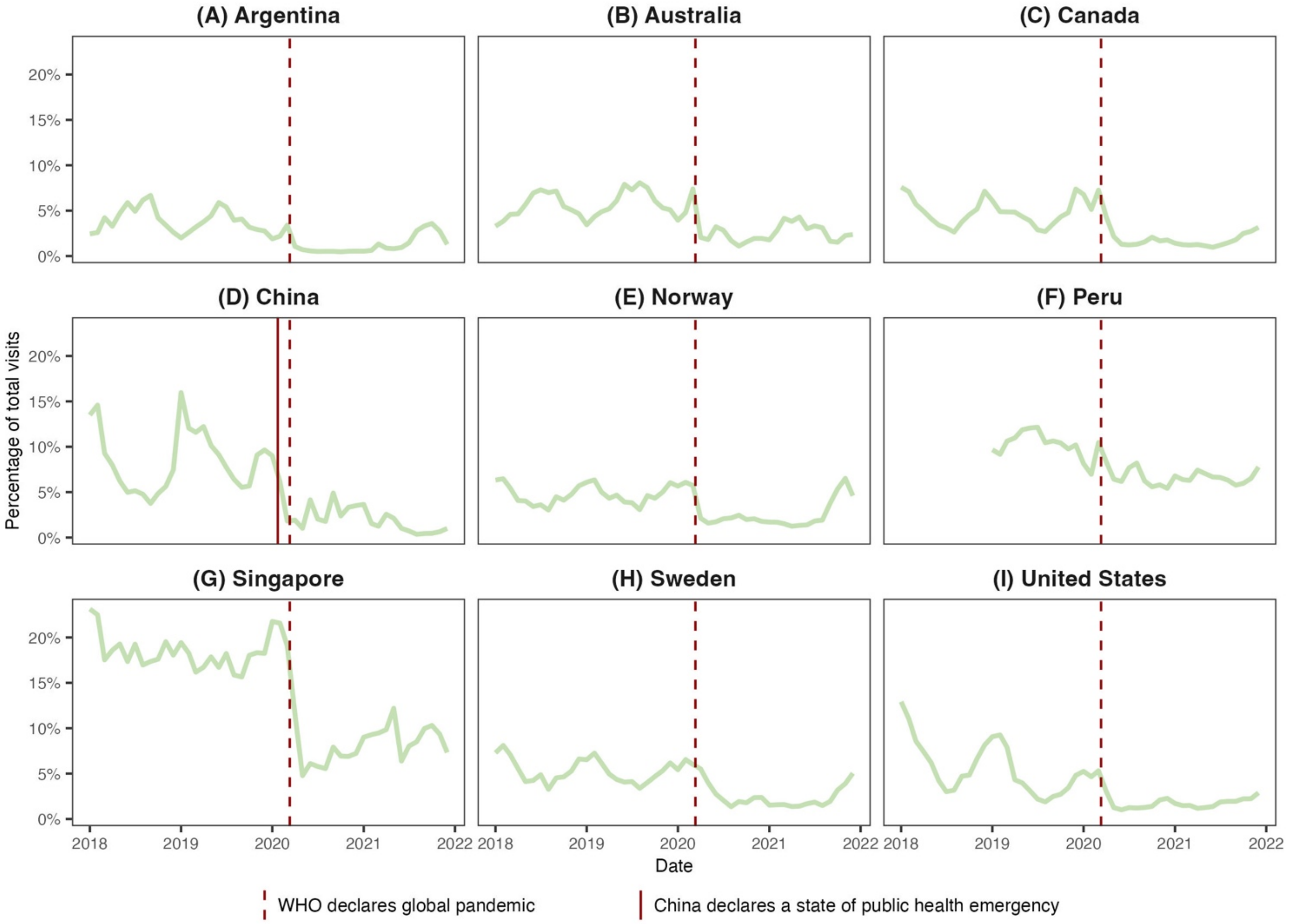

Figure S5. Lower Respiratory Tract Infections (LRTI) visit rates as a percentage of total visits in (A) Argentina, (B) Australia, (C) Canada, (D) China, (E) Norway, (F) Peru, (G) Singapore, (H) Sweden, and (I) United States. Trendlines show the monthly rates of LRTI visits over time. The rates were calculated by dividing the number of LRTI visits by the total visits to primary care services within each month and are expressed as percentages (%).

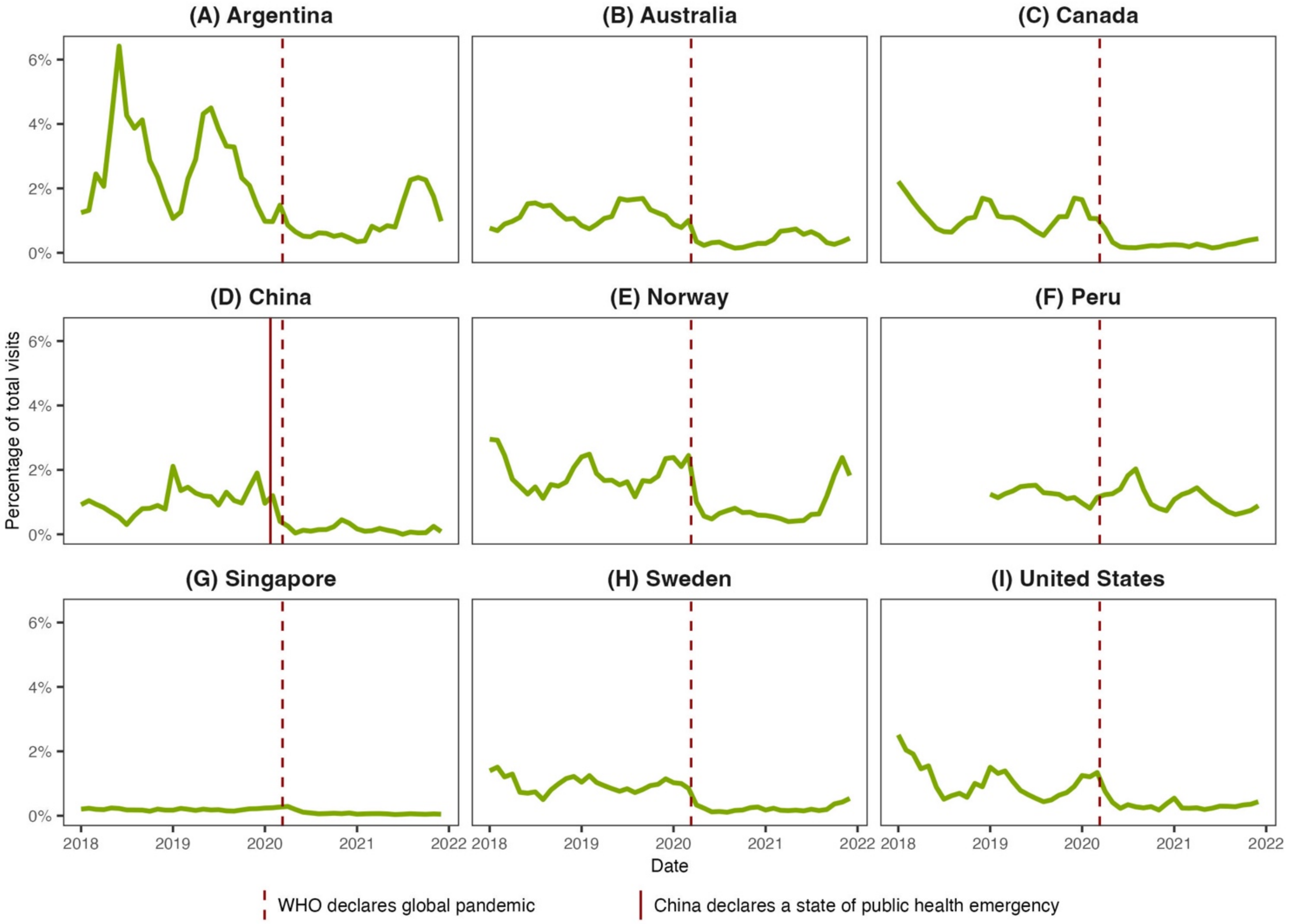

Figure S6. COVID-19 visit rates as a percentage of total visits in (A) Argentina, (B) Australia, (C) Canada, (D) Norway, (E) Peru, (F) Singapore, (G) Sweden, and (H) United States. Trendlines show the monthly rates of COVID-19 visits over time. The rates were calculated by dividing the number of COVID-19 visits by the total visits to primary care services within each month and are expressed as percentages (%).

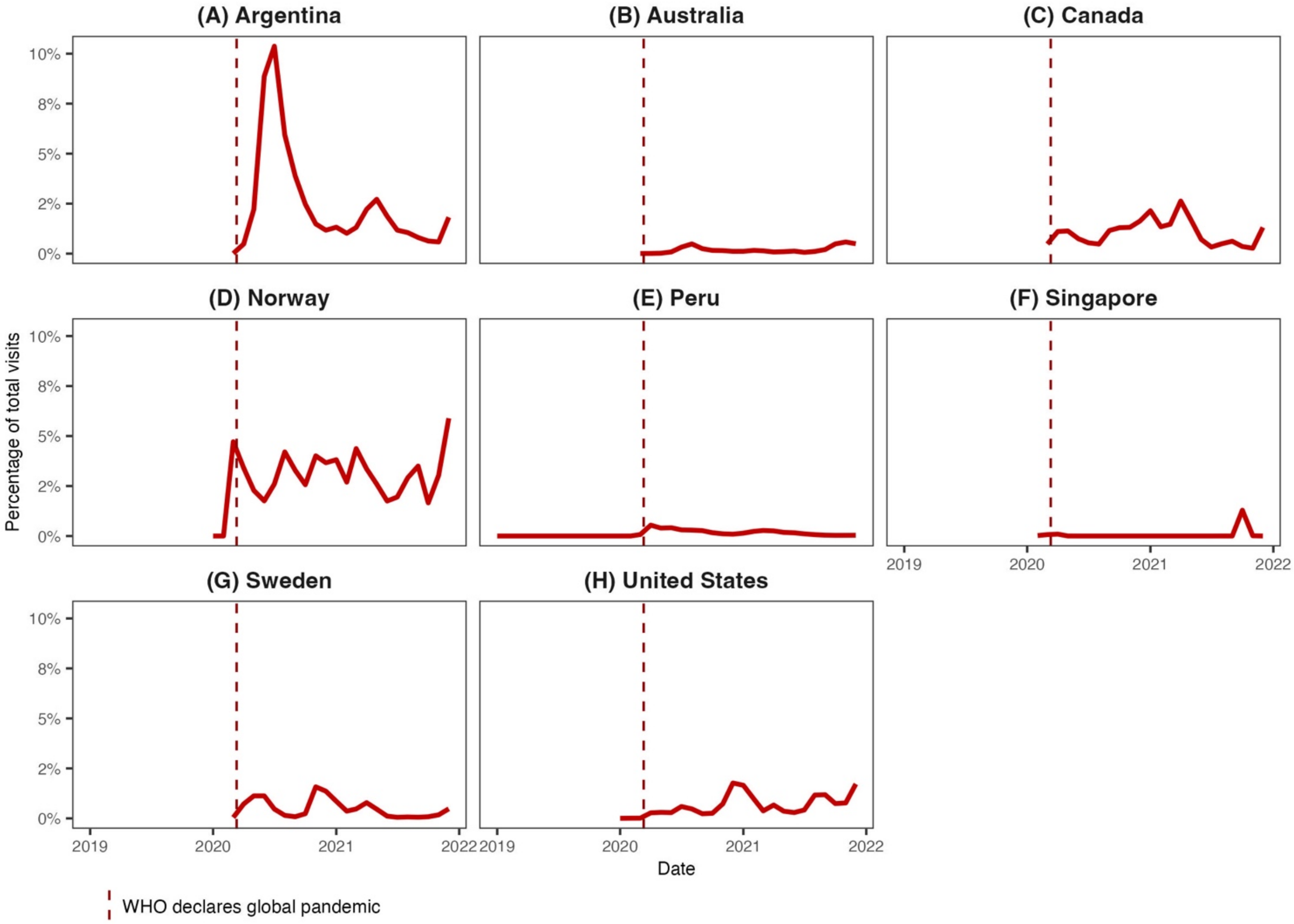

Figure S7. Respiratory allergies visit rates as a percentage of total visits in (A) Argentina, (B) Australia, (C) Canada, (D) China, (E) Norway, (F) Peru, (G) Singapore, (H) Sweden, and (I) United States. Trendlines show the monthly rates of respiratory allergies visits over time. The rates were calculated by dividing the number of respiratory allergies visits by the total visits to primary care services within each month and are expressed as percentages (%).

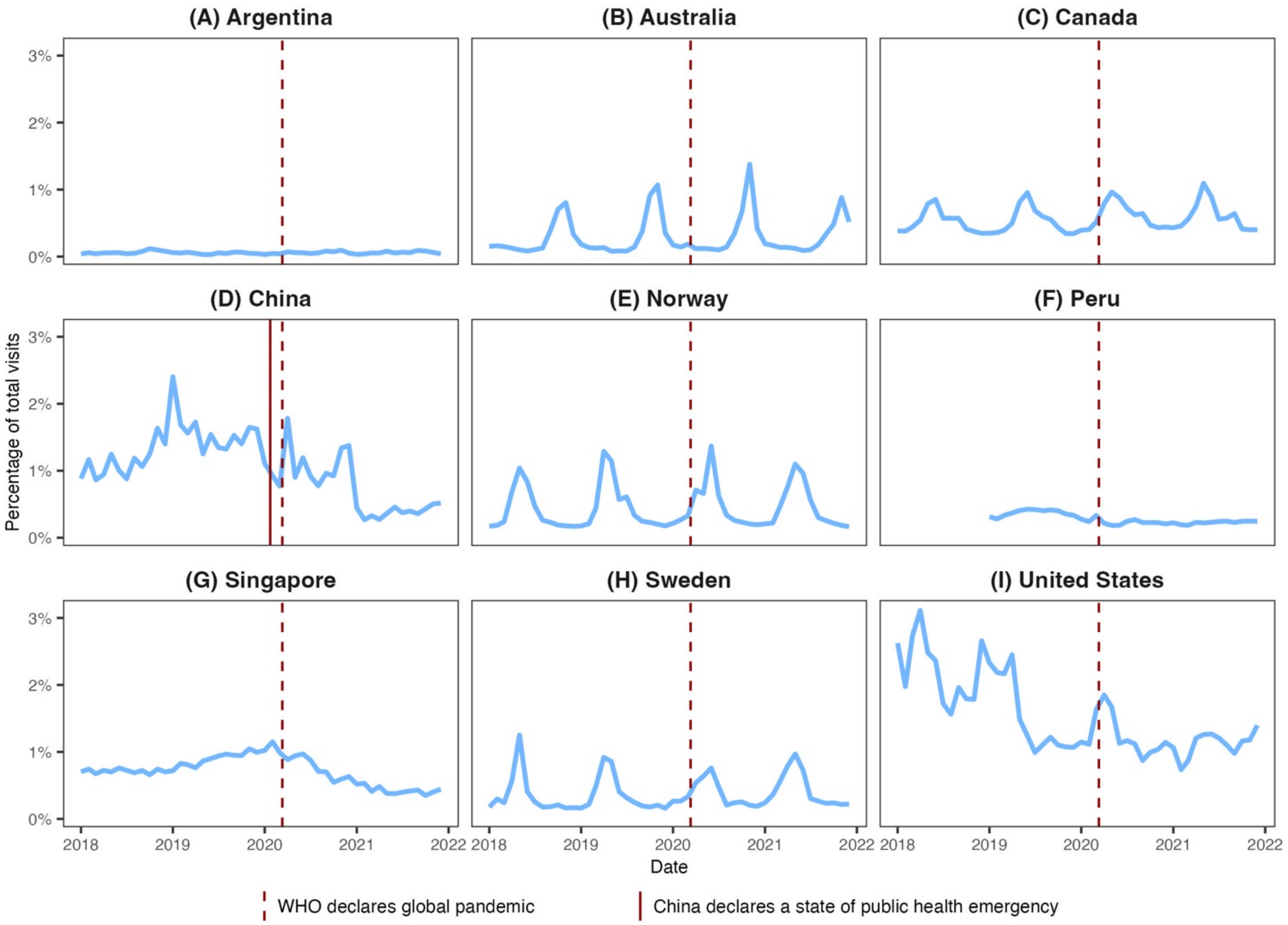

Figure S8. Chronic Obstructive Pulmonary Disease (COPD) visit rates as a percentage of total visits in (A) Argentina, (B) Australia, (C) Canada, (D) China, (E) Norway, (F) Peru, (G) Singapore, (H) Sweden, and (I) United States. Trendlines show the monthly rates of COPD visits over time. The rates were calculated by dividing the number of COPD visits by the total visits to primary care services within each month and are expressed as percentages (%).

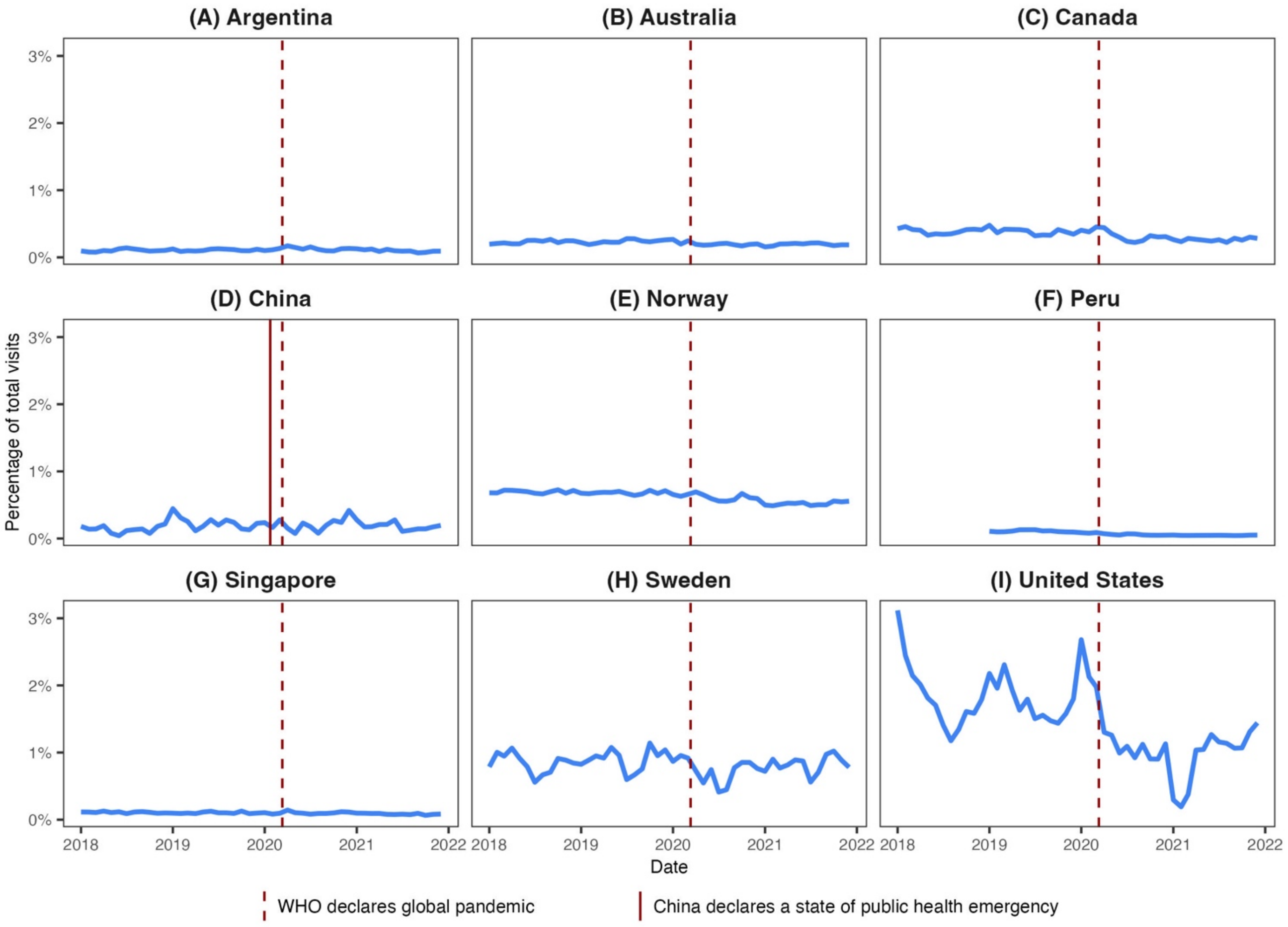

Figure S9. Asthma visit rates as a percentage of total visits in (A) Argentina, (B) Australia, (C) Canada, (D) China, (E) Norway, (F) Peru, (G) Singapore, (H) Sweden, and (I) United States. Trendlines show the monthly rates of asthma visits over time. The rates were calculated by dividing the number of asthma visits by the total visits to primary care services within each month and are expressed as percentages (%).

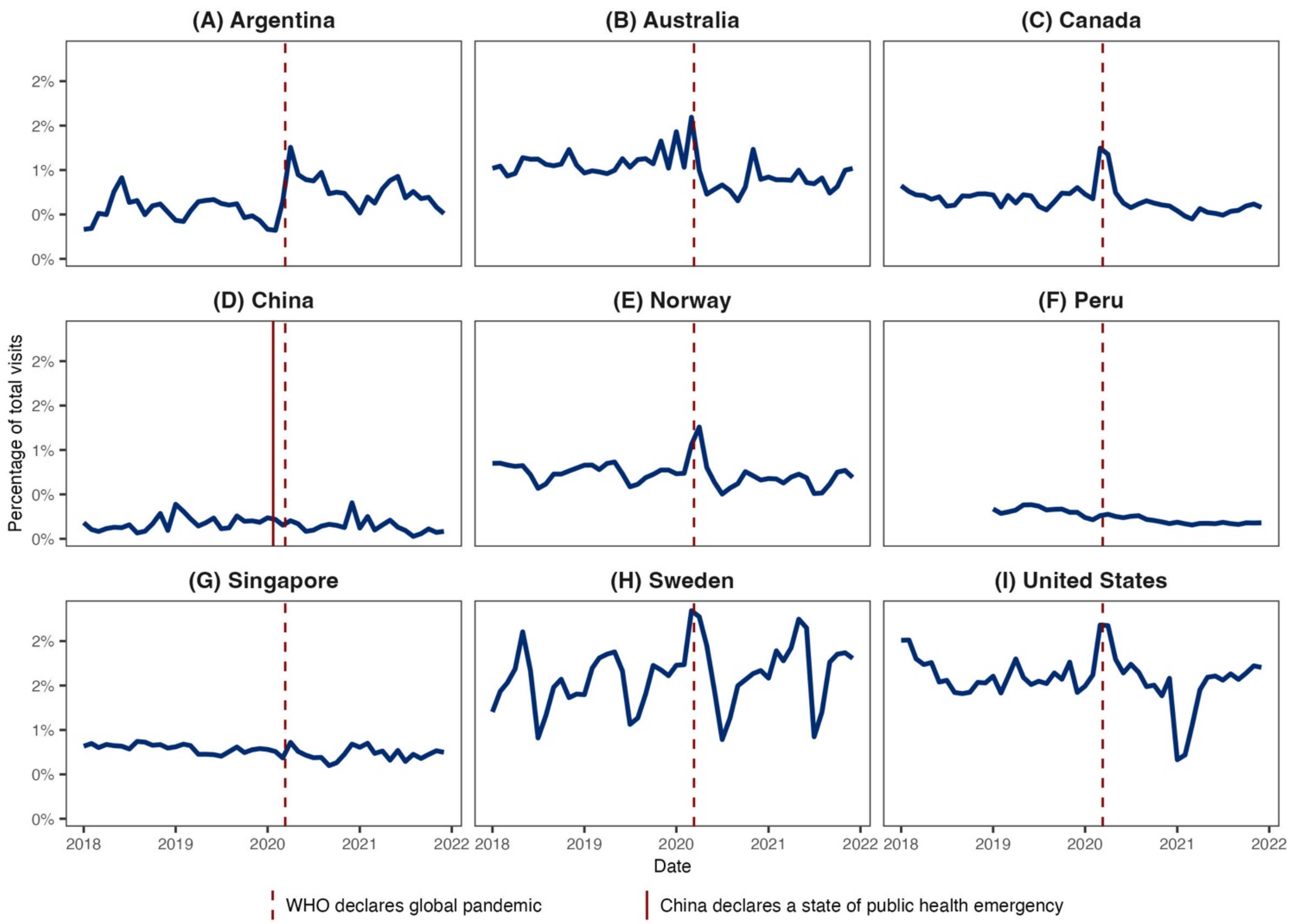

Figure S10. Other respiratory diseases visit rates as a percentage of total visits in (A) Argentina, (B) Australia, (C) Canada, (D) China, (E) Norway, (F) Peru, (G) Singapore, (H) Sweden, and (I) United States. Trendlines show the monthly rates of other respiratory disease visits over time. The rates were calculated by dividing the number of other respiratory disease visits by the total visits to primary care services within each month and are expressed as percentages (%).

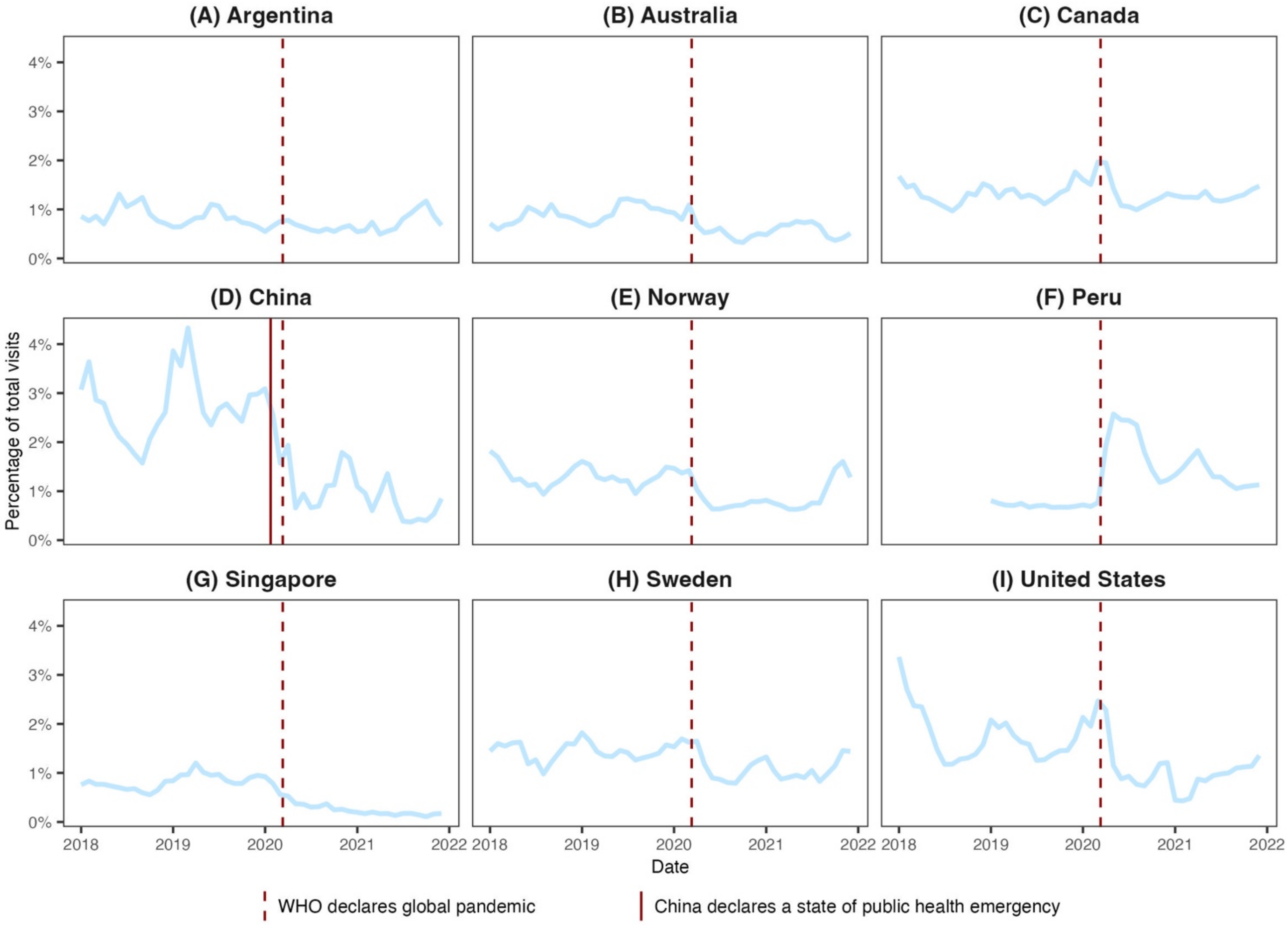

Supplement: Supplementary file 1 [file Data_Sheet_1.pdf]
